# Testing the Acceptability, Feasibility, and Preliminary Efficacy of an Internet-delivered Positive Classroom Management Training (iPCMT) for Elementary School Educators

**DOI:** 10.1007/s10802-026-01453-y

**Published:** 2026-05-12

**Authors:** Antonia L. Boulton, Georgette E. Fleming, Ashneeta H. Prasad, Kelly A. Kershaw, Eva R. Kimonis

**Affiliations:** Parent-Child Research Clinic, School of Psychology, The University of New South Wales, Sydney, NSW Australia

**Keywords:** Conduct problems, Disruptive behavior disorders, Classroom management, Callous-Unemotional traits, Internet-delivered intervention, Schools

## Abstract

Conduct problems emerge early in childhood and can lead to severe maladjustment across the lifespan if not appropriately managed. These problems are highly prevalent in school settings, yet educators reportedly struggle to manage them. When conduct problems co-occur with callous-unemotional (CU) traits, risk for severe, persistent disruptive behavior and academic underachievement increases, necessitating targeted management strategies. The current study evaluated a brief internet-delivered positive classroom management training (iPCMT), informed by the principles and strategies of home- and school-implemented parent management training programs, including those targeting CU traits. Of the 275 elementary school educators that were randomly assigned to either the Immediate-iPCMT condition (*n* = 137) or the Waitlist Control condition (*n* = 138), 228 educators (Mean age = 40.22, 86.1% female-identifying) were included in the final intent-to-treat analyses. At post-intervention, the final educator sample showed significant improvement in their knowledge of conduct problems, but this gain was not maintained for the 28.1% of educators who participated in the 5-week follow-up. Educators who nominated a disruptive student also reported significant decreases in student-educator conflict, but no significant improvements in student-educator closeness, student conduct problems, or student CU traits. Acceptability and feasibility ratings among participants who completed iPCMT were high, but there was a high overall attrition rate. Although iPCMT requires further refinement to increase participant retention and effectiveness, it may serve as a starting point for further research on developing educators’ knowledge of heterogeneous conduct problems. More research is also needed to test the effects of iPCMT in the longer-term, with multi-method outcome measures.

**Supplementary Information:**

The online version contains supplementary material available at 10.1007/s10802-026-01453-y.

Conduct problems refer to a range of persistent behavioral and emotional patterns that violate rules, age-appropriate social norms, and the rights of others (American Psychiatric Association [APA], 2013). These problems are characterized by core symptoms of physical and verbal aggression, anger-irritability, temper tantrums, defiance towards authority figures, deceitfulness, and property destruction (Fairchild et al., 2019). Depending on the frequency and severity of conduct problems, a diagnosis of Oppositional Defiant Disorder (ODD) or Conduct Disorder (CD) may be given, having a worldwide prevalence rate of 5.7% (APA, 2013; Polanczyk et al., 2015). When conduct problems begin before age 10, the risk for long-term adjustment problems increases, including antisocial and violent behavior, relationship dysfunction, academic failure, substance abuse, poor mental and physical health, unemployment, and incarceration (Erskine et al., 2016; Frick et al., 2014; Odgers et al., 2008). In Australia specifically, the prevalence rate of ODD and CD is 5.1% and 2%, respectively, which equates to an estimated total of 287,600 children and adolescents aged 4–17 (Lawrence et al., 2015), therefore placing conduct problems among the most common and challenging mental health concerns faced by Australian schools (Australian Institute of Health & Welfare, 2020). Thus, interventions that enhance educators’ understanding and management of conduct problems are critical.

The most extensively studied and implemented intervention approaches for childhood conduct problems are parent training programs, such as Parent–Child Interaction Therapy (PCIT; Eyberg et al., 1995), Parent Management Training (Barkley, 1997), the Triple P Parenting Program (Sanders, 1999), and Incredible Years (Webster-Stratton, 2005) (see Beelmann et al., 2023 for a review). Indeed, research has long supported the ability of these programs to reduce aggressive and oppositional behavior in children by equipping parents with skills to manage their own stress and emotions more effectively (Webster-Stratton, 1998) and to consistently use positive reinforcement techniques instead of punitive practices (Clark & Frick, 2016). However, there is also strong evidence to suggest that behavioral improvements gained from parenting interventions alone fail to transfer across into school settings (Brotman et al., 2008; Conduct Problems Prevention Research Group, 1999). Accordingly, more calls have been made for the addition of educator training components that cover similar behavior management strategies so that there is consistency across both home and school settings (Brotman et al., 2008; Conduct Problems Prevention Research Group, 1999). With students spending a significant proportion of their time at school, educators are ideally positioned to help deliver the consistency needed for early intervention to succeed, especially with their extensive knowledge about their students’ temperament, strengths, weaknesses, and emotional needs (Yamaguchi et al., 2020).

In order for educators to become agents in preventing students with conduct problems from experiencing an ongoing negative developmental trajectory, it is critical they have access to ongoing, purposeful Professional Development opportunities that help them become attuned to the mental health needs of these students and improve their learning outcomes (Australian Institute for Teaching and School Leadership [AITSL], 2018). The importance of Professional Development is reflected in the Australian Professional Standards for Teachers, which state that all educators at a Proficient, Highly Accomplished, or Lead Teacher level must complete 100 hours of Professional Development across five years to maintain their accreditation (AITSL, 2018). In the most populated Australian state of New South Wales, student/child mental health is one of the five recommended priority areas for Professional Development (NSW Education Standards Authority [NESA], 2024). However, despite there being over 302 NESA-registered Professional Development providers in NSW (NESA, 2025), a sizeable proportion of educators report feeling unprepared and poorly supported in managing conduct problems in their classrooms (Reinke et al., 2011), particularly as they continue to work towards overcoming the ramifications of the coronavirus (COVID-19) pandemic (Pozo-Rico et al., 2020). Indeed, the 2018 Teaching and Learning International Survey (TALIS) found that managing disruptive classroom behavior and being verbally abused by students were among the highest causes of stress in the teaching profession (Ainley & Carstens, 2018). Consequently, as time spent on learning predicts academic achievement (Good et al., 2009), decreased teaching time due to disruptive student behavior may subsequently lead to the declining academic achievement of not just the disruptive students, but also their classmates. Alarmingly, a survey of 1,900 elementary school educators in the United States (US) revealed that almost two and a half hours of learning time are lost each week due to disruptive behavior, adding up to nearly three weeks of lost learning time over the school year (EAB, 2019).

Research suggests that educators’ reported feelings of unpreparedness in managing conduct problems stem from limited training and support (Crum et al., 2016), and may be further exacerbated by the heterogeneity of disruptive behavior presentations that require differentiated management approaches (Cao et al., 2024). That is, a wealth of evidence demonstrates the utility of subtyping children with conduct problems according to the presence of callous-unemotional (CU) traits (Fanti et al., 2013; Pardini & Fite, 2010; Pardini et al., 2012). This research led to the inclusion of the “Limited Prosocial Emotions” specifier in the fifth edition of the *Diagnostic and Statistical Manual of Mental Disorders* (DSM-5; APA, 2013). An estimated one fifth to one quarter of young children with clinically significant conduct problems show this specifier (Neo et al., 2023). CU traits, characterized by a lack of remorse, lack of empathy, insensitivity to punishment, lack of concern about performance, and shallow emotions (Frick et al., 2014), are associated with even more substantial challenges in school settings. Compared to students with conduct problems alone, students with CU traits exhibit more frequent and severe conduct problems, bullying and aggressive behavior (Allen et al., 2018), are less responsive to disciplinary action from educators (Allen et al., 2016), less engaged in school, show lower academic achievement, fewer prosocial behaviors (Levine et al., 2023), and have student-educator relationships that are higher in conflict and lower in closeness (Fleming et al., 2025). This in turn predicts social and academic difficulties, further delinquency, high absenteeism rates and school dropout (Shaw & Shelleby, 2014; Webster-Stratton et al., 2008). Consequently, educators are often expected to manage a heterogeneous group of students with conduct problems using largely uniform behavior management strategies, despite strong evidence that children with elevated CU traits differ in presentation, are less responsive to traditional interventions, and have nuanced intervention needs (Crum et al., 2016; Kimonis et al., 2019).

Existing school-based interventions, including the Incredible Years-Teacher Classroom Management Program (IY–TCM; Webster-Stratton et al., 2008), Teacher–Child Interaction Training (TCIT; Gershenson et al., 2010), the Classroom Organization and Management Program (COMP; Poole & Evertson, 2014), and the Australian-developed ‘Getting On Track In Time’ (Got It!; Plath et al., 2016), have demonstrated effectiveness in improving educators’ skills for supporting disruptive students, particularly in promoting emotional self-regulation and social competence. However, these programs do not typically incorporate the assessment of CU traits or provide explicit guidance on how to recognize them and adapt classroom management strategies for these students (Fleming et al., 2024).

Additionally, many of these school-based interventions rely on intensive, in-person training models involving multi-day workshops and ongoing consultation with trained clinicians or coaches. While effective, such formats place substantial time and logistical demands on educators and require access to specialized personnel, contributing to high implementation costs and limiting scalability. These barriers are particularly salient for under-resourced schools and those in rural or remote settings, where access to specialist support is often constrained (Cohen & Martin, 2023).

To address these barriers to scalability and access, internet-delivered training interventions have gained more attention as a cost-effective means of supporting educators without the need for intensive in-person delivery (Mixon et al., 2019). Another advantage of self-paced, internet-delivered programs is that unlike their face- to-face counterparts, educators can progress through the curriculum according to their own schedule and learning speed (Okonofua et al., 2016). To date, internet-delivered educator training programs have been studied in areas of childhood internalising disorders (i.e., anxiety, depression; Jorm et al., 2010) and eating disorders (McVey et al., 2008). Although these studies produced promising findings, internet-delivered educator training programs have been rarely examined as an intervention for childhood conduct problems (Lochman et al., 2017) and by extension, callous-unemotional traits. Thus, determining whether online Professional Development can effectively enhance the ability of educators to identify and manage heterogeneous disruptive classroom behavior is an important research focus.

## The Current Study

The current study tested the efficacy of “Internet-Delivered Positive Classroom Management Training” (iPCMT), a Professional Development intervention that was directly informed by principles and strategies from PCIT (Niec, 2018), TCIT (Gershenson et al., 2010), the IY–TCM (Webster-Stratton, 2008), and PCIT adapted for children with CU traits (known as PCIT-CU; Kimonis et al., 2019). It was expected that the outcomes of this study would have implications for iPCMT as a potential universal prevention program in elementary schools that also addresses the unique needs of children exhibiting CU traits. An elementary school population was specifically recruited because there is robust evidence that challenging behavior is most malleable under the age of 12 (Loeber et al., 2009). To ensure that educators in rural areas were not deprived from accessing potentially valuable Professional Development resources, as well as to address calls for behavior training programs that do not require substantial time commitments (Duong et al., 2019; Snyder et al., 2011), iPCMT was designed to be self-directed and delivered in a brief (2.5-hour) online format.

The first aim of this study was to examine the feasibility and acceptability of iPCMT as a Professional Development program. We hypothesized that educators who completed iPCMT would rate the program as highly acceptable and feasible at post-intervention, indicated by average scores of at least 75% on the Acceptability of Intervention measure and Feasibility of Intervention Measure.

The second aim of the current study was to test whether iPCMT improves educators’ knowledge of conduct problems, self-efficacy and evidence-based strategy implementation. We hypothesized that educators who completed iPCMT would show significant improvements in knowledge of conduct problems, self-efficacy, and strategy implementation from baseline to post-intervention. We also hypothesized that educators who completed iPCMT would have higher average scores on these measures at the 5-week follow-up, indicative of maintenance of intervention gains.

We also aimed to test whether iPCMT improves educator-reported student conduct problems and CU traits, along with student-educator relationship quality (in terms of closeness and conflict). To achieve this aim, it was important that iPCMT not only focused on conduct problems alone but also targeted CU traits. More specifically, iPCMT includes topics that align with the treatment targets for the CU-specific adaptation of PCIT (PCIT-CU; Kimonis et al., 2019), such as caregiver warmth, rewards-based management strategies, and emotional literacy training. We hypothesized that educators who completed iPCMT would report improvements in student-educator relationship quality and decreases in both student conduct problems and CU traits from baseline to post-intervention.

Finally, we aimed to test group differences by randomly allocating educators into either immediately completing iPCMT or to a Waitlist Control condition. Educators in the Waitlist Control condition were given access to complete iPCMT 11 weeks after the Immediate-iPCMT condition. At post-intervention, it was hypothesized that 1) educators who completed iPCMT would have higher average scores on the knowledge, self-efficacy, and skills implementation measures relative to the Waitlist Control educators; and 2) educators who completed iPCMT would have lower average scores on rater-based measures of disruptive behavior for their nominated student relative to the Waitlist Control educators.

## Method

### Design

A randomized controlled trial was conducted with three repeated measures, including pre-intervention, post-intervention, and a 5-week follow-up. Educators were randomly assigned to either the Immediate-iPCMT Intervention condition or the Waitlist Control condition. While educators in the Waitlist Control condition ultimately had access to iPCMT later in the study period, they had to wait 11 weeks before that access was granted in order to assess the preliminary effects of the intervention.

### Participants

**Fig. 1:**
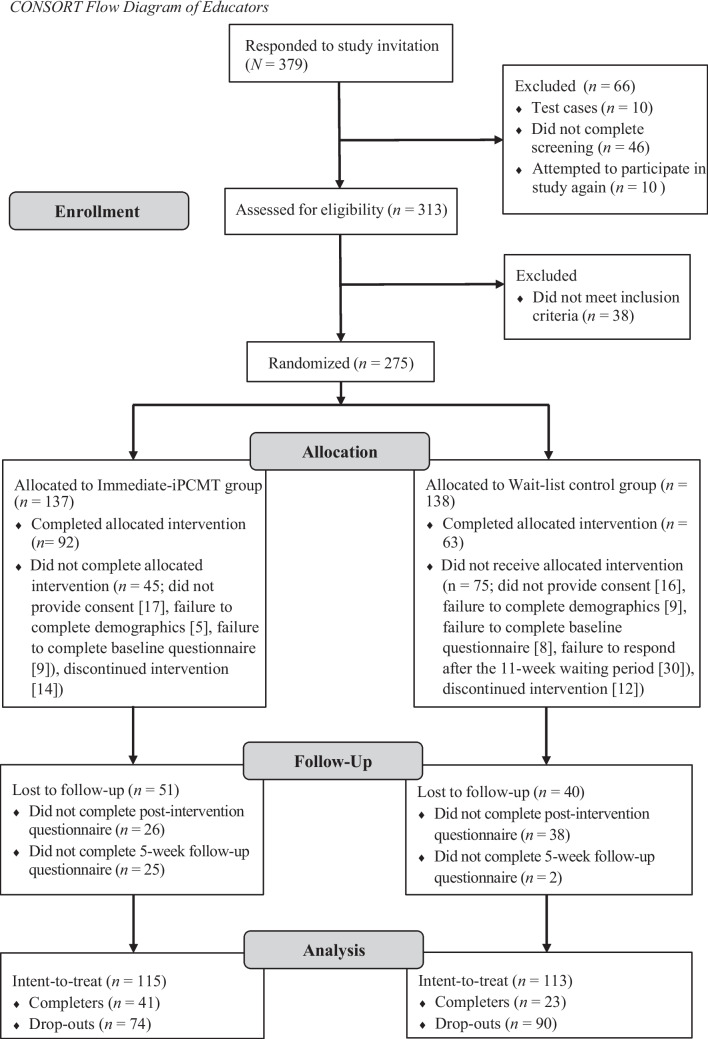
CONSORT flow diagram of educators

A total of *N* = 379 elementary school educators expressed interest in participating in the study. Educators were eligible to participate if they were (1) currently employed as a full-time or casual/part-time elementary school educator, learning support staff member, or paraprofessional in NSW Australia, and (2) not planning to undertake any other training on disruptive behavior management in the next three months. This second eligibility criterion was designed to prevent potentially confounding effects of other behavior management programs. The 275 educators who met these inclusion criteria were randomly assigned to either the Immediate-iPCMT condition (*n* = 137) or the Waitlist Control condition (*n* = 138) automatically via Research Electronic Data Capture (REDCap; Harris et al., 2009; Harris et al., 2019). Figure 1 presents the flow of educators through random allocation, training, and follow-up questionnaires. Recruitment for the current study began in March 2021 (approximately one month after the new school year started) and follow-up questionnaires were completed by February 2024. Out of the 275 eligible educators, 228 [aged 21–70 years (M = 40.22, SD = 12.51)] completed the demographic questionnaire at baseline. The intervention was completed by 155 (Immediate-iPCMT condition, *n* = 92; Waitlist Control condition [after the 11-week waiting period], *n* = 63) of the 228 educators. Educators who completed all the questionnaires and iPCMT modules were entered into a draw to win $100 for their school. The sample size of the current study satisfied the recommended target (*N* = 50; 25 participants per condition) for feasibility studies (Lancaster & Williamson, 2004; McIntosh et al., 2018; Thabane et al., 2010).

### Procedure

The current study was approved by the University of New South Wales Human Research Approval Panel (3511/3275). Educators were recruited via email invitations that provided information about the study and a link to the REDCap web platform that hosted the study. Upon accessing the REDCap platform, educators were screened and presented with a downloadable Participant Information Statement and Consent Form. After each educator was deemed eligible to participate and had provided their informed consent, they were asked to nominate one of their own disruptive students about which to respond on the subsequent questionnaires. This was done by having the educators enter in their nominated student’s initials before commencing the demographic questionnaire. If an educator was unable to identify a specific student with conduct problems, they responded to the questionnaires about a hypothetical disruptive student depicted in a vignette.

All participating educators then spent approximately 40 min completing a pre-intervention (baseline) questionnaire that included demographic questions, after which they received an email that differed depending on their assigned experimental condition. The email received by the Immediate-iPCMT condition contained the link to commence their iPCMT video modules, whereas educators in the Waitlist Control condition were instructed to wait 11 weeks for their access link. To create the point of comparison needed to examine between-group differences, the educators in the Waitlist Control condition were given a repeat of the baseline questionnaire at the end of the 11-week waiting period, from which they could then commence the four iPCMT modules.

The duration of the iPCMT modules, designed to equip educators with comprehensive and up-to-date information on the management of heterogeneous conduct problems, was approximately 2.5 hours. Table 1 provides an overview of the contents of iPCMT modules. Given the self-paced nature of the modules, educators were not required to complete them all in one sitting, provided the modules were completed within 10 days. Immediately after completing the iPCMT modules, educators were prompted to complete a post-intervention questionnaire that contained the same measures as the baseline questionnaire but with the addition of the acceptability and feasibility measures. To fully complete their participation in the study, each educator was asked to complete a much shorter set of questionnaires (approx. 20 min in length) at the five-week post-intervention mark.

**Table 1:** Objectives and topics covered in each iPCMT module

Module	Objective	Topics Covered
1	Identify different types and causes of disruptive student behaviors	a) Introduction (4m15s) b) Background in severe behavioral problems ○ What are Behavior Problems? (16m55s) ○ **What are Limited Prosocial Emotions? (7m58s)** c) Fundamentals to understanding child behavior ○ Functions and principles of disruptive behavior (7m10s) ○ Determining the function of problem behaviors (8m35s) ○ Power of attention (5m25s) ○ Understanding the coercive cycle (5min12sec)
2	Apply strategies proven to enhance student-educator relationships	a) P.R.I.D.E. Skills ○ Praise (13m55s) ○ Reflection (2 min) ○ Imitation (2m15s) ○ Description (5m25s) ○ **Emotional expression (2m58s)** b) Avoiding commands (2m25s) c) Avoiding unnecessary questions (4m55s) d) Avoiding negative talk (3m30s) e) **Using rewards to promote desired behaviors (9m10s)** f) **Promoting emotional literacy in students (20m38s)** g) Delivering clear directions (3m40s)
3	Effectively respond to disruptive and aggressive student behavior	a) Planned ignoring, and praising the positive opposite (10m50s) **b) Validating, redirecting, and following through (3m56s)** c) Sit and Watch time (12m15s)
4	Teach students emotional literacy and emotion regulation skills	**a) Tailoring strategies for students with Behavior Problems and Limited Prosocial Emotions (8m30s)**

*Note.* Topics specifically relating to CU traits are indicated in bold

## Measures

### Demographics

At baseline, educators were asked to provide information on their sex, gender identity, age, race, geographic location, and employment status. They also provided information about their teaching career, such as the sector they teach in, their accreditation status, and years of teaching experience. Given that iPCMT was designed to support educators, no demographic information was collected about nominated students.

### Knowledge of Conduct Problems

We assessed educator knowledge of the different subtypes of conduct problems with a study-specific 35-item *Knowledge Test* created by three clinical psychologists with expertise in research and treatment of childhood conduct problems and CU traits. Educators answered multiple-choice questions about the content covered in the iPCMT intervention, such as the characteristics of conduct problems (e.g. “Which if the following symptoms are all associated with conduct problems?”) and CU traits (e.g. “Which of the following options is NOT a relevant target behavior for students with Limited Prosocial Emotions?”), evidence-based principles of behavior management (e.g. “What are some of the ways adults accidentally reinforce or maintain problematic behavior?”), and evidence-based behavior management strategies (e.g. “Which of the following is the best example of a clear direction?”). Knowledge test scores were calculated as the number of correct responses and ranged between 0 and 35, with higher scores indicating greater knowledge about childhood conduct problems. The internal consistency of knowledge test scores ranged from acceptable to excellent from pre-intervention to follow-up (Cronbach’s α = .71-.91, McDonald’s ω = .71-.91). See Supplementary Table 1 for a list of the 35 Knowledge Test questions, including the topic areas they fall under.

### Perceived Self-Efficacy

We assessed educator self-efficacy using the 4-item Classroom Management subscale from the *Teacher’s Sense of Self-Efficacy Scale – Short Form* (TSSE-SF; Tschannen-Moran & Hoy, 2001). Educators rated their self-efficacy (e.g. “How much can you do to control disruptive behavior in the classroom?”) on a 9-point Likert scale from 1 (*None at all*) to 9 (*A Great Deal*)*.* In the present study, the internal consistency of self-efficacy scores was good to excellent from pre-intervention to follow-up (Cronbach’s α = .86–92, McDonald’s ω = .86-.92). Scores ranged from 4–36, with higher scores indicating greater self-efficacy.

### Perceived Student-Educator Relationship Quality

We assessed student-educator relationship quality using the 15-item *Student–Teacher Relationship Scale – Short Form* (STRS-SF; Pianta, 2001)*.* Educators rated their relationship with their nominated student according to levels of closeness (e.g. “This student values their relationship with me”) and conflict (e.g. “Dealing with this student drains my energy”) on a 5-point Likert scale from 1 (*Definitely does not apply*) to 5 (*Definitely applies*). Scores on the 8-item Closeness subscale range from 8–40 and the 7-item Conflict subscale scores range from 7–35, with lower scores on the Closeness subscale and higher scores on the Conflict subscale indicating lower student-educator relationship quality. For the Closeness subscale, the internal consistency was good to excellent from pre-intervention to follow-up (Cronbach’s α = .84–90, McDonald’s ω = .85–90) and the Conflict subscale showed good internal consistency at all time-points (Cronbach’s α = .81-.82, McDonald’s ω = .81-.83).

### Conduct Problems

We assessed the perceived conduct problems of educator-nominated students using the 5-item Conduct Problem subscale from the educator-report version of the *Strengths and Difficulties Questionnaire* (SDQ-CP; Goodman, 2001). Educators rated the level of their nominated student’s conduct problems (e.g. “Often loses temper”) on a 3-point Likert scale from 0 (*Not true*) to 2 (*Certainly true*). Scores range from 0–10, with higher scores indicating more severe conduct problems. The severity of conduct problems was contexualized according to Mellor’s (2005) Australian mean SDQ-CP score of 1.0 (SD = 1.5) and the four accompanying scoring categories, including 0–2 (Close to average), 3 (Slightly raised), 4–5 (High), and 6–10 (Very high). SDQ-CP scores demonstrated questionable to acceptable internal consistency from pre-intervention to follow-up (Cronbach’s α = .68-.78, McDonald’s ω = .70-.79).

### Callous-Unemotional Traits

We assessed the perceived CU traits of educator-nominated students using the 8-item abbreviated educator version of the *Inventory of Callous-Unemotional Traits* (ICU; Frick, 2004; Kimonis et al., 2015). Educators rated the extent of their nominated student’s CU traits (e.g. “Is concerned about the feelings of others”) on a 4-point Likert scale from 0 (*Not true at all*) to 3 (*Definitely true*), with scores ranging from 0–24. All 8 positively worded items were reverse coded in a negative direction, such that higher scores indicated greater CU traits. In the present study, the internal consistency of ICU scores was good to excellent from pre-intervention to follow-up (Cronbach’s α = .87-.93, McDonald’s ω = .87-.93).

### Frequency and Usefulness of Classroom Management Strategies

We utilized a study-specific *Strategy Implementation Survey* (SIS) developed by the three clinical psychology researchers to measure how frequently educators used seven evidence-based recommended classroom management strategies covered in iPCMT (such as “Planned ignoring”, “Validating and redirecting”, and “Rewarding appropriate behavior”) over the past five weeks. Educators were also asked to rate how often they used five other strategies that are not typically recommended in evidence-based approaches for conduct problems (such as “Detentions”, “School suspensions”, and “Removal of stickers/tokens for misbehavior”). See Supplementary Table 2 for a list of the SIS items. The SIS was then re-administered to measure how useful educators found each recommended and non-recommended strategy. Educators rated the frequency and usefulness of both recommended and non-recommended strategies on a 4-point Likert scale from 0 (*Never*) to 4 (*Very often*), with scores ranging from 0–28 and 0–20, respectively. Higher scores on each subscale indicated that educators often implemented these strategies into their practice and found them helpful. In terms of the recommended strategies, the internal consistency of the Frequency subscale was questionable to acceptable from pre-intervention to follow-up (Cronbach’s α = .69-.73, McDonald’s ω = .71-.73). For the corresponding Usefulness subscale, the internal consistency was good to excellent from pre-intervention to follow-up (Cronbach’s α = .85-.93, McDonald’s ω = .85-.93). Regarding the non-recommended strategies, the internal consistency of the Frequency subscale was within the questionable to acceptable range from pre-intervention to follow-up (Cronbach’s α = .65-.74, McDonald’s ω = .65-.75). For the corresponding Usefulness subscale, the internal consistency was questionable to good from pre-intervention to follow-up (Cronbach’s α = .69-.83, McDonald’s ω = .71-.87).

### Acceptability

We assessed the acceptability of iPCMT as a Professional Development program using the 5-item Acceptability of Intervention Measure (AIM; Weiner et al., 2017). Educators rated acceptability (e.g. “This training meets my approval”) on a 5-point Likert scale from 1 (*Completely disagree*) to 5 (*Completely agree*). In the present study, the AIM was only administered at the post-intervention time-point and the total scores had excellent internal consistency (Cronbach’s α = .95, McDonald’s ω = .95).

### Feasibility

We assessed the feasibility of iPCMT as a Professional Development program using the 4-item Feasibility of Intervention Measure (FIM; Weiner et al., 2017). Educators rated feasibility (e.g. “The skills taught in this program seem implementable”) on a 5-point Likert scale from 1 (*Completely disagree*) to 5 (*Completely agree*). Similar to the AIM, the FIM was only administered at post-intervention and the total scores had excellent internal consistency (Cronbach’s α = .94, McDonald’s ω = .94). See Supplementary Table 3 for an expanded list of the reported reliability coefficients for each outcome measure.

### Planned Analyses

All analyses were conducted using an intent-to-treat (ITT) approach, which included all participants who commenced the baseline questionnaire, irrespective of whether they completed that baseline questionnaire, the iPCMT modules, or the questionnaires at post-intervention or follow-up. Data analysis was also originally planned to span across four time-points, including baseline (pre-intervention), post-intervention, 5-week follow-up, and 10-week follow-up. However, the 10-week follow-up questionnaire had a very low response rate for both conditions (*n* = 30; 23 in the Immediate-iPCMT condition and 7 in the Waitlist Control condition) and was therefore excluded from analyses.

Descriptive statistics were used to evaluate the educators’ mean scores for the two brief intervention acceptability and feasibility measures.

Linear Mixed-Effects Modelling was conducted using data from the full intent-to-treat sample to evaluate iPCMT’s effectiveness over time (pre-intervention, post-intervention, and 5-week follow-up), irrespective of condition assignment. The Maximum Likelihood Estimation (MLE) method was used to account for missing data. Six continuous educator-reported outcomes were evaluated, including knowledge of conduct problems, self-efficacy, the frequency of both recommended and non-recommended classroom management skills, and the usefulness ratings of both recommended and non-recommended classroom management skills. Additionally, two continuous student-related outcomes were evaluated, including student-educator closeness and student-educator conflict. Within-subject change was examined by including the fixed effect of Time (weeks since baseline). Since there is little consensus on the correct approach to calculating effect sizes from mixed model analyses (Luo & Furukawa, 2023), effect sizes for within-subject differences were calculated by applying the following suggested formula (Feingold, 2009) to each outcome measure: ([Model-Adjusted Mean Change_Post-intervention_ – Model-Adjusted Mean Change_Pre-intervention_] / Pooled SD of raw scores) and ([Model-Adjusted Mean Change_5-Week Follow-up_ – Mean Change_Post-intervention_] / Pooled SD of raw scores]. To control the family-wise error rate (FWER) for multiple within-group tests of change over time, Holm-Bonferroni corrections (Holm, 1979) were applied. Since three time comparisons were examined (pre-intervention to post-intervention, post-intervention to 5-week follow-up, and pre-intervention to 5-week follow-up), corrections were applied separately for the educator-reported outcomes (six measures assessed across three time comparisons, totaling to 18 comparisons) and the student-related outcomes (two measures assessed across three time comparisons, totaling to six comparisons).

Linear Mixed Effects Modelling was also used to assess the effects of Intervention (iPCMT versus no iPCMT) and Time (pre-intervention and post-intervention) on two educator-reported outcomes, including knowledge of conduct problems and self-efficacy. Four student-related outcomes were also evaluated, including student-educator closeness and student-educator conflict, along with perceived student conduct problem severity and CU traits. To allow time-matched comparisons between conditions, questionnaire responses from the Immediate-iPCMT condition at post-intervention were compared against Waitlist Control responses to the repeated baseline questionnaire that was administered after the 11-week waiting period. Additionally, since vignette-based responses were not considered reflective of observed student behavior, only the educators who nominated a real student were included in the analyses pertaining to student-educator relationship, and student-level conduct problems and CU traits. Linear and quadratic change were examined by including the fixed effects of Time (weeks since baseline) and the interaction of Time x Condition as predictors. Effect sizes for between-subject differences were calculated by applying the following suggested formula (Feingold, 2009) to each outcome measure at pre-intervention and post-intervention: [Model-Adjusted Mean Change_Immediate-iPCMT_ – Model-Adjusted Mean Change_Waitlist Control_] / Pooled SD of raw scores]. Holm-Bonferroni corrections were applied to control the family-wise error rate for multiple between-group tests. Since differences between two groups (Immediate-iPCMT and Waitlist Control) were examined from pre-intervention to post-intervention, corrections were applied separately for the educator-reported outcomes (two measures assessed for two groups across one time comparison, totaling to four comparisons) and the student-related outcomes (four measures assessed for two groups across one time comparison, totaling to eight comparisons). Condition x Time interaction effects were also corrected using the Holm-Bonferroni method, with corrections conducted separately for the same two educator-reported outcomes and four student-related outcomes.

Effect sizes were rated according to Cohen’s (1988) guidelines: trivial (< 0.2), small (0.2–0.49), medium (0.50–0.79), or large (≥ 0.80). All analyses were conducted with IBM SPSS Statistics (Version 30; IBM Corp, 2024) and alpha levels were set at *p* < .05.

## Results

### Educator Characteristics and Baseline Differences

The characteristics of the educators who completed the demographics portion of the baseline questionnaire are reported in Table 2**.** Whitney-Mann U tests found no significant difference between the Immediate-iPCMT and Wait-list Control conditions for age. Chi-square tests found no significant differences between conditions in categorical variables such as gender (majority female-identifying, 86.8%), race (majority White Australian, 72.4%), employment status (majority permanent full-time, 49.3%), accreditation status (majority Proficient, 66.1%), years of teaching experience (fairly even distribution of early, mid, and established), and geographic location (majority metropolitan, 89.5%). Fisher’s Exact test found no significant differences between conditions in the type of school (majority working in Government schools, 96.5%) and institution setting (almost all primary/elementary, 99.6%). Additionally, no significant differences were found between the conditions on any dependent measures at baseline. The only significant difference between conditions at baseline was for the educators’ current role (majority teachers, 69.2%), χ2(1) = 4.51, *p* = .034. However, we did not control for this variable in subsequent analyses since the question about current role was introduced later in the study and only 45% of the 228 educators had responded to it.

**Table 2:** Demographic characteristics of educators for total sample and training group allocation

Variable	Total Sample	Immediate-iPCMT	Waitlist Control	Test Statistics
	*M (SD)*	*M (SD)*	*M (SD)*	*t or* χ2
Educator Age (years)	*N* = 228 40.22 (12.51)	*n* = 115 39.60 (12.46)	*n* = 113 40.86 (12.57)	*t*(226*)* = −0.76, *p* = .45
	*N (%)*	*n (%)*	*n (%)*	
Gender	*N* = 204	*n* = 100	*n* = 104	χ2(1) = 0.26, *p* = .61 ^a^
Woman	177 (86.8)	88 (88.0)	89 (85.6)	
Man	26 (12.7)	11 (11.0)	15 (14.4)	
Prefer not to answer	1 (0.5)	1 (1.0)	0 (0.0)	
Race / Ethnicity	*N* = 228	*n* = 115	*n* = 113	χ2(1) = .91, *p* = .34 ^b^
White	165 (72.4)	80 (69.6)	85 (75.2)	
Asian	21 (9.2)	14 (12.2)	7 (6.2)	
Pacific Islander	2 (0.9)	0 (0.0)	2 (1.8)	
Middle Eastern	14 (6.1)	9 (7.8)	5 (4.4)	
Aboriginal or Torres Strait Islander	6 (2.6)	1 (0.9)	5 (4.4)	
African	2 (0.9)	2 (1.7)	0 (0.0)	
Other	18 (7.9)	9 (7.8)	9 (8.0)	
Current Role	*N* = 104	*n* = 52	*n* = 52	χ2(1) = 4.51, *p* = .034*^c^
Teacher	72 (69.2)	31 (59.6)	41 (78.8)	
Learning Support Staff or Paraprofessional	20 (19.2)	12 (23.1)	8 (15.4)	
Other	12 (11.5)	9 (17.3)	3 (5.8)	
Years Experience	*N* = 227	*n* = 114	*n* = 113	χ2(1) = 0.71, *p* = .40 ^d^
0–5 years (early)	78 (34.4)	42 (36.8)	36 (31.9)	
6–15 years (mid)	76 (33.5)	37 (32.5)	39 (34.5)	
16–30 + years (established)	73 (32.2)	35 (30.7)	38 (33.6)	
Accreditation Status	*N* = 227	*n* = 114	*n* = 113	χ2(1) = 0.11, *p* = .74 ^e^
Unaccredited	14 (6.2)	9 (7.9)	5 (4.4)	
Conditionally accredited	15 (6.6)	7 (6.1)	8 (7.1)	
Provisionally accredited	45 (19.8)	20 (17.5)	25 (22.1)	
Proficient	150 (66.1)	78 (68.4)	72 (63.7)	
Highly accomplished	2 (0.9)	0 (0.0)	2 (1.8)	
Lead	1 (0.4)	0 (0.0)	1 (0.9)	
Employment Status	*N* = 227	*n* = 114	*n* = 113	χ2(1) = 0.12, *p* = .73 ^f^
Permanent full-time	112 (49.3)	55 (48.2)	57 (50.4)	
Permanent part-time	18 (7.9)	9 (7.9)	9 (8.0)	
Temporary full-time	67 (29.5)	37 (32.5)	30 (26.5)	
Temporary part-time/Casual	28 (12.3)	13 (11.4)	15 (13.3)	
Other	2 (0.9)	0 (0.0)	2 (1.8)	
Geographic Location	*N* = 228	*n* = 115	*n* = 113	χ2(1) = 0.23, *p* = .63 ^g^
Metropolitan	204 (89.5)	104 (90.4)	100 (88.5)	
Regional	21 (9.2)	10 (8.7)	11 (9.7)	
Rural	3 (1.3)	1 (0.9)	2 (1.8)	
Type of School	*N* = 227	*n* = 114	*n* = 113	*p* = .07 ^h^
Government/public	219 (96.5)	107 (93.9)	112 (99.1)	
Systemic (e.g., Catholic)	2 (0.9)	1 (0.9)	1 (0.9)	
Independent/Non-government	5 (2.2)	5 (4.4)	0 (0.0)	
Other	1 (0.4)	1 (0.9)	0 (0.0)	
Institution Setting	*N* = 226	*n* = 114	*n* = 113	*p* = .99 ^i^
Early childhood	1 (0.4)	1 (0.9)	0 (0.0)	
Primary/Elementary	225 (99.6)	113 (99.1)	112 (100.00)	

*Note.* *p < .05. M = Mean. SD = Standard Deviation

^a^Gender binarized (1 = Woman; 2 = Man, Non-Binary) for chi-squared test

^b^Race binarized (1 = White, 2 = Asian, Middle Eastern, Aboriginal or Torres Strait Islander, African, Pacific Islander, Other) for chi-squared test

^c^Current Role binarized (1 = Teacher, 2 = Learning Support, Paraprofessional) for chi-squared test

^d^Years experience binarized (1 = Early, 2 = Mid, Established) for chi-squared test

^e^Accreditation Status binarized (1 = Unaccredited, Conditionally accredited, Provisionally accredited, 2 = Proficient, Highly accomplished, Lead) for chi-squared test

^f^Employment Status binarized (1 = Permanent full-time, Temporary full-time, 2 = Permanent part-time, Temporary part-time/Casual) for chi-squared test

^g^Geographic location binarized (1 = Metropolitan, 2 = Rural, Regional) for chi-squared test

^h^Type of School (1 = Government/Public, 2 = Systemic, Independent/Non-government, Other) for Fisher’s Exact test

^i^Institution Setting (1 = Primary/Elementary, 2 = Early childhood) for Fisher’s Exact test

The characteristics of educators who dropped out (i.e. drop-outs) versus those who completed all stages of the study (i.e. completers) were also examined and are reported in Supplementary Table 4. Chi-square tests found no significant differences between drop-outs and completers in gender, race, employment status, geographic location, and current role. Additionally, Fisher’s Exact test found no significant differences regarding the type of school and institution setting. However, we found significant group differences in age, condition, years of teaching experience, and accreditation status, indicating that the drop-outs tended to be younger (t(226) = 2.46, *p* = .02), have an earlier career (χ2(1) = 6.16, *p* = .01) and lower accreditation status (χ2(1) = 6.12, *p* = .01), and allocated in the Waitlist Control condition (χ2(1) = 6.61, *p* = .01).

### Acceptability and Feasibility

Table 3 presents results for the Acceptability of Intervention Measure (AIM) and the Feasibility of Intervention Measure (FIM), including frequencies for each item and total mean scores. Quantitative feedback from the educators who completed the post-intervention questionnaire was generally positive. Mean scores for AIM items were all above the scale midpoint (Ms = 3.65–3.79), as were mean scores for FIM items (Ms = 3.94–4.11). For the AIM, the item regarding the educators’ approval of iPCMT received the highest ratings, with 53.3% endorsing a rank of 4 (“Agree”) and 16.0% endorsing a rank of 5 (“Completely Agree”). For FIM, the item pertaining to the possibility of applying the skills covered in iPCMT was rated the highest, with 63.1% of educators endorsing a rank of 4 (“Agree”) and 18.1% endorsing a rank of 5 (“Completely Agree”). Overall, iPCMT received an average of 18.59 (74.36%) out of a possible score of 25 for acceptability and an average of 16.30 (80.15%) out of a possible score of 20.

**Table 3:** Mean ratings and Standard Deviations (SD) for acceptability and feasibility of intervention items

Item		Rating Frequency (%)					Mean score
**AIM**	*N*	**1**	**2**	**3**	**4**	**5**	**M (SD)**
This training meets my approval	150	1 (0.7)	8 (5.3)	37 (24.7)	80 (53.3)	24 (16.0)	3.79 (.80)
This training is appealing to me	150	4 (2.7)	9 (6.0)	37 (24.7)	76 (50.7)	24 (16.0)	3.71 (.90)
I like this training program	150	5 (3.3)	5 (3.3)	48 (32.0)	71 (47.3)	21 (14.0)	3.65 (.88)
I welcome this training program	149	2 (1.34)	7 (4.7)	38 (25.5)	78 (52.3)	24 (16.1)	3.77 (.82)
I am satisfied with this training program	150	4 (2.7)	12 (8.0)	36 (24.0)	76 (50.7)	22 (14.7)	3.67 (.92)
AIM Total	150						18.59 (3.93)
**FIM**	*N*	**1**	**2**	**3**	**4**	**5**	**M (SD)**
The skills taught in this program seem implementable	149	0 (0.0)	1 (0.7)	18 (12.1)	94 (63.1)	36 (24.2)	4.11 (.62)
Successfully managing disruptive student behavior using the strategies I learned from this program seems possible	149	0 (0.0)	3 (2.0)	25 (16.8)	94 (63.1)	27 (18.1)	3.97 (.66)
Applying the strategies I learned from this program to students in my classroom seems doable	149	0 (0.0)	0 (0.0)	25 (16.8)	97 (65.1)	27 (18.1)	4.01 (.59)
The skills I learned in this program seem easy to use	149	0 (0.0)	1 (0.7)	33 (22.1)	39 (26.2)	26 (17.4)	3.94 (.65)
FIM Total	149						16.3 (2.31)

*Note.* AIM = Acceptability of Intervention Measure; FIM = Feasibility of Intervention Measure. Each item is rated on a scale of 1 to 5. A ranking of 1 is ‘Completely disagree’, while a ranking of 5 is ‘Completely agree’. Higher scores indicate higher acceptability and feasibility of iPCMT. M = Mean. SD = Standard Deviation. *N* = Number of educators who responded to each item within the AIM and FIM

### Effects of iPCMT Over Time

Table 4 summarizes the results of the Linear Mixed-Effects Modelling. The raw means and standard deviations for the overall educator sample can be found in Supplementary Table 5.

**Table 4:** Adjusted mean differences and standard errors for overall educator sample across time

Outcome Variable	Pre-intervention to Post-intervention				Post-intervention to 5WFU				Pre-intervention to 5WFU			
	Mean Difference [95% CI] ^b^	SE	*p*	*p*_adj_	Mean Difference [95% CI] ^c^	SE	*p*	*p*_adj_	Mean Difference [95% CI] ^d^	SE	*p*	*p*_adj_
Knowledge	2.85 [2.02, 3.48]	.51	** < .001**	** < .001**	−4.02 [−5.33, −2.71]	.67	** < .001**	** < .001**	−1.17 [−2.46, .11]	.65	.074	.740
Self-Efficacy	.62 [.08, 1.17]	.28	**.026**	.286	-.94 [−1.69, -.18]	.38	**.015**	.180	-.32 [−1.07, .43]	.38	.403	1.000
Student-Educator Relationship Closeness^a^	.50 [-.10, 1.10]	.31	.104	.416	-.58 [−1.41, .26]	.42	.173	.519	-.08 [-.90, .75]	.42	.853	.853
Student-Educator Relationship Conflict ^a^	−1.12 [−1.88, -.36]	.38	**.004**	**.024**	.19 [-.86, 1.24]	.53	.720	1.000	-.93 [−1.96, .10]	.52	.076	.380
Frequency of Recommended Classroom Management Strategies	.11 [-.54, .76]	.33	.735	1.000	.16 [-.74, 1.06]	.46	.735	1.000	.27 [-.62, 1.16]	.45	.555	1.000
Utility of Recommended Classroom Management Strategies	1.71 [.86, 2.57]	.43	** < .001**	**.001**	.45 [-.74, 1.65]	.61	.457	1.000	2.16 [.99, 3.34]	.60	** < .001**	**.005**
Frequency of Non-Recommended Classroom Management Strategies	-.22 [-.64, .21]	.22	.319	1.000	-.08 [-.67, .51]	.30	.787	.787	-.30 [-.87, .28]	.29	.317	1.000
Utility of Non-Recommended Classroom Management Strategies	.63 [-.11, 1.36]	.37	.095	.855	1.70 [.66, 2.73]	.53	**.001**	**.013**	2.32 [1.31, 3.34]	.52	** < .001**	** < .001**

*Note.* 5WFU = 5-week follow-up assessment. Unadjusted *p*-values are presented alongside Holm-Bonferroni adjusted *p*-values (*p*_adj_) for within-group comparisons over time. Corrections were conducted separately for educator-reported outcomes and student-related outcomes

^a^ Only inclusive of participants who nominated a disruptive student by providing their initials at the beginning of the study. 164 participants within the intent-to-treat sample provided student initials

^b^ Mean difference = Post-intervention – Pre-intervention

^c^ Mean difference = Post-intervention – 5-week follow-up

^d^ Mean difference = 5-week follow-up – Pre-intervention

As shown in Table 4, the estimated marginal means (i.e. model adjusted means) indicated that educator knowledge of conduct problems significantly improved from pre-intervention (M = 20.66, SE = .41) to post-intervention (M = 23.51, SE = .45; *d* = .58), which remained significant following correction. However, knowledge test scores significantly deteriorated from post-intervention to the 5-week follow-up (M = 19.49, SE = .61; *d* = -.62), falling below baseline. This deterioration also remained significant following correction. A similar pattern emerged for educator self-efficacy, in which there was a significant improvement from pre-intervention (M = 29.49, SE = .31) to post-intervention (M = 30.11, SE = .33; *d* = .15), and a significant decrease at the 5-week follow-up (M = 29.17, SE = .42; d = -.22) that fell below baseline. However, these self-efficacy results did not remain significant following corrections.

Regarding the skill implementation measures, usefulness ratings for recommended classroom management strategies significantly increased from pre-intervention (M = 17.96, SE = .39) to post-intervention (M = 19.67, SE = .42; *d* = .37), and this remained significant following correction. There was a trend towards improvement from post-intervention to the 5-week follow-up, but this did not reach significance. Additionally, although usefulness ratings of non-recommended classroom management strategies did not significantly increase from pre-intervention (M = 8.09, SE = .35) to post-intervention (M = 8.72, SE = .38), there was a significant increase from post-intervention to the 5-week follow-up (M = 10.42, SE = .52; *d* = .39) that remained significant after correction. However, there were no significant differences in the frequency of recommended and non-recommended classroom management strategies at any time-point.

In terms of the student-educator relationship subscales, there was a trend towards improvement in perceived closeness from pre- to postintervention, along with slight deterioration at the 5-week follow-up, but these differences did not reach significance. On the other hand, perceived conflict scores significantly improved (i.e. decreased) from pre-intervention (M = 23.68, SE = .52) to post-intervention (M = 22.56, SE = .54; *d* = -.19), which remained significant following correction, but there was no significant difference at the 5-week follow-up (M = 22.75, SE = .65).

### Pre- to Post-Intervention Evaluation

Table 5 summarizes the results of the Linear Mixed-Effects Modelling comparing the Immediate-iPCMT and Waitlist Control conditions, and Supplementary Table 6 presents the raw means and standard deviations for both conditions. It is important to note that out of the 228 educators who provided baseline data, 164 educators (Immediate-iPCMT, n = 87; Waitlist Control, n = 77) nominated a disruptive student when they began participating in the study. The remaining 64 educators that responded to a hypothetical student vignette instead of identifying a disruptive student were included in analyses for all the teacher-related outcomes, but were excluded from the analysis of student-related outcomes, including student-educator relationship, child conduct problems, and CU traits.

**Table 5:** Adjusted means differences and standard errors for the immediate-iPCMT and waitlist control conditions

Outcome Variable	Immediate-iPCMT				Waitlist Control			
	Mean Difference [95% CI] ^b^	SE	*p*	*p*_adj_	Mean Difference [95% CI] ^b^	SE	*p*	*p*_adj_
Knowledge	3.21 [2.26, 4.16]	.48	** < .001**	** < .001**	-.07 [−1.0, .86]	.47	.875	.875
Self-Efficacy	.85 [.23, 1.46]	.31	**.007**	**.021**	-.27 [−1.0, .46]	.37	.466	.932
Student-Educator Relationship Closeness ^a^	.17 [-.46, .81]	.32	.585	1.000	.54 [-.70, 1.78]	.62	.384	1.000
Student-Educator Relationship Conflict ^a^	−1.36 [−2.26, -.46]	.45	**.004**	**0.032**	-.95 [−2.21, .31]	.63	.136	.816
Student Conduct Problems ^a^	-.10 [-.48, .29]	.19	.619	1.000	.08 [-.42, .59]	.25	.742	1.000
Student Callous-Unemotional Traits ^a^	-.78 [−1.43, -.13]	.32	**.019**	.133	.06 [-.87, .98]	.46	.901	.901

*Note.* Unadjusted *p*-values are presented alongside Holm-Bonferroni adjusted *p*-values (*p*_adj_) for between-group comparisons. Corrections were conducted separately for educator-reported outcomes and student-related outcomes

^a^ Only inclusive of participants who nominated a disruptive student by providing their initials at the beginning of the study. 164 participants within the intent-to-treat sample provided student initials

^b^ Mean difference = Post-intervention – Pre-intervention

As shown in Table 5, the estimated marginal means (i.e. model adjusted means) indicated that in the Immediate-iPCMT condition, knowledge of conduct problems significantly improved from pre-intervention (M = 20.83, SE = .42) to post-intervention (M = 24.04, SE = .46), with a medium effect size (*d* = .72). This improvement in knowledge remained significant after correction. There were also significant improvements in self-efficacy (M = 29.67, SE = .39 at pre-intervention; M = 30.52, SE = .41 at post-intervention; *d* = .21), student-educator conflict (M = 23.96, SE = .62 at pre-intervention; M = 22.60, SE = .65 at post-intervention; *d* = -.25), and perceived student CU traits (M = 26.14, SE = .46 at pre-intervention; M = 25.36, SE = .48 at post-intervention; *d* = -.19). However, only the improvements in self-efficacy and student-educator conflict remained significant after correction. Although the Immediate-iPCMT condition showed a trend towards improvement in student-educator relationship closeness and perceived student conduct problems, these pre- to post-intervention differences did not reach statistical significance. On the other hand, the Waitlist control condition showed a trend towards deterioration on all outcome measures except for student-educator closeness, but none of those pre- to post-intervention differences reached statistical significance. As shown in Table 6A-B, there were significant Condition x Time interaction effects on knowledge of conduct problems (*β* = 3.34*, p* < .01) and self-efficacy (*β* = 1.11*, p* < .05), with medium (*d* = .75) and small (*d* = .28) effect sizes respectively. Both interaction effects remained significant after correction.

**Table 6:** Linear mixed-effects models estimating the impact of iPCMT on outcome variables from pre-intervention to post-intervention

**Fixed Effects**	***β***	***SE***	***p***	***p***_**adj**_	***β***	***SE***	***p***	***p***_**adj**_	***β***	***SE***	***p***	***p***_**adj**_
**Predictor**	**A. Knowledge**				**B. Self-Efficacy**				**C. Student-Educator Closeness** ^a^			
Intercept	19.97	.78	** < .001**		29.95	.60	** < .001**		29.77	.95	** < .001**	
Condition	−2.34	1.10	**.035**		−1.13	.84	.182		2.18	1.29	.091	
Time	.001	.49	.998		-.26	.35	.459		.66	.49	.179	
Condition x Time	3.19	.66	** < .001**	** < .001**	1.11	.48	**.021**	**.021**	-.51	.65	.436	1.000
**Random Effects**	***σ***^***2***^	**SE**	***p***		***σ***^***2***^	**SE**	***p***		***σ***^***2***^	**SE**	***p***	
Subject (Intercept)	9.36	1.66	** < .001**		11.00	1.37	** < .001**		29.45	3.77	** < .001**	
Residual	9.93	1.10	** < .001**		4.90	.53	** < .001**		5.84	.77	** < .001**	
**Model Fit**												
−2LL	2262.67				2015.28				1563.00			
AIC	2274.67				2027.28				1575.00			
BIC	2298.50				2050.91				1596.48			
**Fixed Effects**	***β***	***SE***	***p***	***p***_**adj**_	***β***	***SE***	***p***	***p***_**adj**_	***β***	***SE***	***p***	***p***_**adj**_
**Predictor**	**D. Student-Educator Conflict** ^a^				**E. Student Conduct Problems** ^a^				**F. Student Callous-Unemotional Traits** ^a^			
Intercept	25.38	1.00	** < .001**		6.38	.41	** < .001**		25.73	.73	** < .001**	
Condition	-.06	1.35	.963		.38	.55		1.000	1.19	.99	.229	
Time	-.94	.57	.102		.08	.24		1.000	.05	.42	.914	
Condition x Time	-.42	.75	.582	1.000	-.18	.31	.566	1.000	-.83	.55	.134	1.000
**Random Effects**	***σ***^***2***^	**SE**	***p***	***σ***^***2***^	**SE**	***p***	***σ***^***2***^	***SE***	***p***			
Subject (Intercept)	23.38	3.26	** < .001**	3.79	.54	** < .001**	12.86	1.79	** < .001**			
Residual	8.05	1.06	** < .001**	1.36	.18	** < .001**	4.16	.55	** < .001**			
**Model Fit**												
−2LL	1575.52				1090.69				1387.62			
AIC	1587.52				1102.69				1399.62			
BIC	1609.00				1124.12				1421.00			

*Note. β* = Estimate of fixed effect, SE = Standard Error, *σ*^*2*^ = variance*.* Unadjusted *p*-values are presented alongside Holm-Bonferroni adjusted *p*-values (*p*_adj_) for Condition x Time interaction effects. Corrections were conducted separately for educator-reported outcomes and student-related outcomes

^a^ Only inclusive of participants who nominated a disruptive student by providing their initials at the beginning of the study. 164 participants within the intent-to-treat sample provided student initials

## Discussion

The importance of addressing conduct problems among young children in school settings is well established in both empirical research (Whitley et al., 2013) and policy reports (NESA, 2017; EAB, 2019). Despite this recognition, educators consistently report feeling overwhelmed and ill-prepared to manage disruptive behavior in the classroom, citing gaps in knowledge, skills, confidence, and access to appropriate resources (Whitley et al., 2013). These challenges appear to be compounded by the heterogeneity of conduct problem presentations, including subgroups of students who differ in symptom profiles and responsiveness to standard classroom management strategies. As a result, existing Professional Development opportunities often provide educators with broadly applicable behavior management techniques but limited guidance on how to recognize and respond to students whose conduct problems are accompanied by elevated CU traits. In this context, the intervention evaluated in the current study was developed to address educators’ expressed training needs by increasing knowledge of conduct problem heterogeneity and providing accessible, scalable support for tailored classroom behavior management.

The purpose of the current study was to examine the preliminary effects of iPCMT, a brief, online, self-guided training intervention designed to equip elementary school educators and learning support staff with knowledge about the different subtypes of child conduct problems and evidence-based skills for managing their corresponding behaviors in the classroom. The results of this study lend partial support for the efficacy of iPCMT, as it demonstrated an immediate positive effect on educator knowledge around conduct problems. However, this improvement was not sustained among the minority of educators (28.1%) in the intent-to-treat sample who returned to complete the 5-week follow-up questionnaire. Additionally, the current study did not find sufficient evidence to suggest that educators who completed iPCMT experienced significant increases in the frequency of using recommended evidence-based classroom management skills, although educators who completed the follow-up reported these skills to be increasingly useful across time.

In terms of participant evaluation, feasibility ratings of iPCMT exceeded the 75% benchmark whereas acceptability ratings just fell short, suggesting that educators found that the skills covered were implementable with their students, but the current online training delivery could be improved.

In regard to changes in the perceived problems of educator-nominated students with conduct problems, our hypothesis regarding the Immediate-iPCMT condition predicting lower scores on the student behavior outcome measures (i.e. SDQ-CP and ICU) relative to the Waitlist Control condition was not supported, as the observed significant improvement in student levels of CU traits was lost following the application of a Holm-Bonferroni correction.

### Implications for Educators

Previous research has explored the efficacy of school-based educator training interventions, finding that they had moderate to large effects on educators’ knowledge, self-efficacy, and implementation of recommended classroom management strategies in both the short- and long-term (Webster-Stratton, 2008). While findings from the current study reported a significant improvement in educator knowledge following iPCMT, this was not maintained in the longer-term (i.e., 5-weeks post-intervention) among the minority of educators who completed the follow-up questionnaire. Additionally, the significant improvement in self-efficacy found within the overall intent-to-treat sample was lost following Holm-Bonferroni correction. Past research has similarly found that one-off training interventions tend to be insufficient for creating long-lasting, sustainable change in educators’ knowledge and use of recommended classroom management strategies (O’Reilly et al., 2018). Indeed, Darling-Hammond and colleagues (2024) recognized that due to the complexity of addressing student behavior, educators may need recurrent training to have adequate mental health literacy and competency to manage disruptive students. Alternatively, given its brief and accessible format, iPCMT may function most effectively as a universal component within a stepped-care or multi-tiered intervention model, alongside more intensive school-based adaptations of PCIT that provide targeted educator coaching to support implementation of PCIT and PCIT-CU strategies with students presenting with elevated conduct problems (Fleming et al., 2026).

Furthermore, several strategies could be undertaken to improve the effectiveness of iPCMT, such as developing and introducing a brief revision course on the most important concepts covered—in a similar way to conventional First Aid training providers who offer ‘refresher’ courses on important emergency procedures such as Cardiopulmonary Resuscitation (Jorm et al., 2010); providing online seminars that bridge the gap between learning and implementing the skills covered in iPCMT; in vivo coaching to implement strategies with disruptive students in the classroom context; and/or communities of practice. Future iterations of iPCMT might also benefit from active learning exercises to facilitate educator engagement, retention, and real-world application of its content, which might also bolster intervention acceptability ratings. Additional content addressing educator factors that can reduce capacity to implement behavioral management skills (e.g. stress management or mindfulness training: Braun et al., 2020; Herman & Reinke, 2014) may also enhance outcomes.

### Strengths and Limitations

Few studies have evaluated educator-focused interventions that target disruptive student behaviors (Cao et al., 2024). Thus, the current study builds on this prior scant literature base in finding that participation in iPCMT positively influenced educators’ knowledge around heterogeneous conduct problems. A major strength of the current study was the online delivery format of iPCMT. Indeed, integrating video technology into classroom management training tends to increase the perceived practical value of Professional Development in classroom management (Birman et al., 2000), which was made evident in the current study by the high average ratings on the Feasibility of Intervention Measure from the participating educators. A previous feasibility study recommended that the effectiveness of Professional Development programs for educators may be enhanced by offering knowledge in easily digestible doses and ensuring that it is accessible and affordable (Woods, 2014). The current study adopted both recommendations in its use of video technology to provide educators across NSW (including rural and remote areas) with the opportunity to learn current, evidence-based strategies for behavior management at no cost to them and to progress through the iPCMT curriculum at a pace that suited their own learning speed. This flexible, self-paced learning is difficult to achieve in lengthy face-to-face Professional Development programs, which can inundate educators with information without permitting enough time to process it. Another central strength of the current study was its inclusion of a Waitlist Control condition and randomized trial design, which enabled us to assess the effect of iPCMT against receiving no intervention.

Notwithstanding these strengths, there were some study limitations that must be considered when interpreting results. First, the random assignment of educators to a condition was conducted at the individual level without stratification by school, as ethical approval to collect identifiable school-level information was not obtained until later in the study. Therefore, although participants were drawn from at least 36 different schools, the potential for school-level clustering and condition contamination cannot be ruled out. Second, since data were collected using only educator-report questionnaires, they are subject to mono-method (questionnaires only) and mono-informant (single reporter) bias. Further work is needed to test the efficacy of iPCMT using multi-method and multi-informant measurement of child outcomes, such as parent-report measures and observational measures of child conduct problems.

Third, of the 228 educators included in the intent-to-treat sample, only 155 educators (68%) went on to complete iPCMT, and retention at the 5-week follow-up was lower (41% of those who completed iPCMT). Attrition of this magnitude is common in research evaluating self-directed online interventions, particularly when participants are not actively seeking treatment (Sanders et al., 2012). In addition, individual reimbursement or incentives for educator participation were not permitted under the State Education Research and Partnerships (SERAP) ethics approval process. The absence of individual-level incentives may therefore have further contributed to reduced participant retention. Beyond these factors, the magnitude and pattern of attrition in this study introduce several other interpretative considerations. As seen previously in Fig. 1, the attrition rate of the Waitlist Control condition (90/138 participants; 65%) was noticeably higher than that of the Immediate-iPCMT condition (74/137 participants; 54%), which could be attributed to the lengthy 11-week waiting period resulting in a loss of motivation to engage with the intervention. Chi-square analysis confirmed that this difference was statistically significant (Supplementary Table 4), suggesting that missing data may not be missing-at-random. Indeed, the higher attrition in the Waitlist Control condition may have reduced between-group differences by selectively retaining participants with greater baseline motivation. Moreover, for student-related outcomes, which were derived from the smaller subgroup of educators who nominated a student, selective attrition may have further reduced representativeness and precision of estimates. Sensitivity analyses, such as multiple imputation or pattern-mixture modelling, were not conducted in the current study and therefore represent an important direction for future trials with larger samples and more complete follow-up data. Accordingly, findings should be interpreted carefully and as preliminary, especially the findings at follow-up. Thus, future research on iPCMT could adopt a different experimental design, whereby educators are allocated into either receiving iPCMT or a more generic Professional Development program.

Additionally, there were many educators who started iPCMT, but stopped shortly after starting. This may be because the iPCMT intervention did not have any interactive components since it was delivered online instead of using face-to-face coaching, activities, and feedback. Rather, the iPCMT intervention consisted of watching online videos. Although these videos were highly informative and evidence-based, their didactic lecture format may have affected educator engagement. The literature suggests that interactive engagement with content can help promote transfer of knowledge into practice, even when facilitated with technology (Lochman et al., 2017). For example, future iterations of iPCMT could include online interactive components such as quizzes, discussion forums, or video feedback on performance. Since keeping educators engaged in iPCMT is imperative to achieving gains, future research could investigate whether adding interactive components improves participant retention. Expanding on the limitation of attrition, educators were given the flexibility to complete iPCMT in one sitting or across multiple sessions, yet data on the number and duration of sessions per educator were not systematically recorded. Future research should therefore incorporate more in-depth activity logs to evaluate the influence of pacing on participant engagement. Despite the high attrition rates seen in the current study, using Hierarchical Linear Modelling (in conjunction with the Maximum Likelihood Estimate method) to predict the effects of iPCMT on each outcome measure helped compensate for this issue, as many common statistical methods such as Multivariate Analysis of Variance (MANOVA) and Generalized Linear Models do not account for missing data. It is also important to note that post-hoc power estimates varied across outcomes, as power was high for outcomes with significant Condition x Time interaction effects (educator knowledge and self-efficacy; 99% and 56%, respectively) and much lower for non-significant interaction effects (ranging from 8 – 24%). Since these estimates depended on observed effect, they should be interpreted with caution and indicate the need for replication with a larger sample. Another limitation is that the internal consistency of the SDQ-CP was relatively low when assessed using Cronbach’s alpha, most likely due to the small number of items. Contrastingly, McDonald’s omega provided acceptable reliability estimates, and while this pattern is consistent with previous psychometric evaluations of the SDQ subscales (Sanders et al., 2012; Stone et al., 2015), the limitations associated with Cronbach’s alpha should be considered when interpreting the results involving conduct problems*.* Similar consideration should be applied to results involving strategy implementation, as some subscales within the SIS were also in the questionable range, which could be due to the SIS being study-specific. A final limitation is that the findings may not generalize to other, more diverse populations of educators. In acknowledging these limitations, the current study still offers useful insights into potential approaches to the effective delivery of Professional Development in classroom management of child conduct problems and guidance for future research.

## Conclusion

In summary, the results of the current study revealed that following a 2.5-hour online, self-guided intervention, educators experienced significant short-term improvement in their knowledge of conduct problems and reported levels of student-educator conflict, but these gains were not maintained at follow-up. Short-term improvements in reported self-efficacy and student CU traits were also observed amongst the educators who were allocated into the Immediate-iPCMT condition, but these improvements were not significant following Holm-Bonferroni corrections. While the uptake of recommended evidence-based classroom management strategies also improved with intervention, these improvements were not large enough to reach statistical significance. Nevertheless, the results are valuable since research in the area of developing educators’ competencies around heterogeneous conduct problems is limited (Whitley et al., 2013). By addressing conduct problems proactively, educators can create an environment where all students can thrive academically and emotionally. While iPCMT clearly requires further refinement to enhance its effectiveness for improving both educator and student outcomes, it offers a promising starting point in undertaking the task of providing educators with evidence-based training about conduct problems.

## Supplementary Information

Below is the link to the electronic supplementary material.

Supplementary file1 (DOCX 108 KB)
