# Supplementary material for: Testing the Acceptability, Feasibility, and Preliminary Efficacy of an Internet-delivered Positive Classroom Management Training (iPCMT) for Elementary School Educators
Source: Res Child Adolesc Psychopathol. 2026 May 12;54(3):70. doi: 10.1007/s10802-026-01453-y (PMC13167873; doi:10.1007/s10802-026-01453-y)
Supplement: Supplementary file 1 — Supplementary file1 (DOCX 108 KB) [file 10802_2026_1453_MOESM1_ESM.docx]

**Supplementary Materials**

**Supplementary Table 1**

*Knowledge Test Items, and Percentage that Each Answer was Endorsed by Total Educator Sample*

| **Item** | **Question** | **Answer** | **Frequency (%)** | | |
| --- | --- | --- | --- | --- | --- |
| *Topic: Characteristics of conduct problems* | | | Pre | Post | 5WFU |
| 1 | Select the correct statement below (choose one) | Genetic factors (e.g., family history of mental health concerns) are the main causes for why certain children display severe behaviour problems | 3 (1.6%) | 1 (0.6%) | 2 (2.7%) |
|  |  | ​​Environmental factors (e.g., home environment) are the main causes for why certain children display severe behaviour problems | 11 (5.8%) | 6 (3.9%) | 2 (2.7%) |
|  |  | **Multiple genetic and environmental factors interact with one another to make it more likely that children display severe behavioural problems** | **168 (88.4%)** | **142 (91.6%)** | **64 (87.7%)** |
|  |  | None of the above statements are correct | 8 (4.2%) | 6 (3.9%) | 5 (6.8%) |
| 2 | Which of the following symptoms are all associated with Conduct Problems? | Lack of remorse/guilt, Lack of empathy, poor social skills, hyperactivity, verbal and physical aggression | 31 (16.3%) | 10 (6.5%) | 12 (16.9%) |
|  |  | Defiance towards authority figures, hostility, non-compliance, hyperactivity/impulsivity, verbal and physical aggression | 85 (44.7%) | 60 (38.7%) | 29 (40.8%) |
|  |  | Lack of remorse/guilt, Lack of empathy, Shallow/superficial emotions, uncaring attitudes about performance | 10 (5.3%) | 15 (9.7%) | 12 (16.9%) |
|  |  | **Defiance towards authority figures, hostility, non-compliance, deceitfulness/lying, verbal and physical aggression** | **64**  **(33.7%)** | **70 (45.2%)** | **18 (25.4%)** |
| 27 | Select the statement that is INCORRECT: | Higher emotional literacy is associated with improved academic functioning | 30 (16.4%) | 20 (13.2%) | 10 (14.5%) |
|  |  | Higher emotional literacy is associated with improved social functioning | 8 (4.4%) | 4 (2.6%) | 4 (5.8%) |
|  |  | **Emotional literacy naturally increases and develops over time at similar rates for all children** | **128 (69.9%)** | **117 (77.0%)** | **47 (68.1%)** |
|  |  | Emotional literacy helps children to understand, communicate and regulate their emotions | 17 (9.3%) | 11 (7.2%) | 8 (11.6 %) |
| *Topic: Callous-unemotional (CU) traits / limited prosocial emotions (LPE)* | | |  | | |
| 3 | Which of the following symptoms are associated with Limited Prosocial Emotions | Lack of remorse/guilt, Lack of empathy, poor social skills, hyperactivity, verbal and physical aggression | 62 (32.6%) | 31 (20.0%) | 19 (26.8%) |
|  |  | Defiance towards authority figures, poor social skills, non-compliance, hyperactivity, verbal and physical aggression | 23 (12.1%) | 15 (9.7%) | 9 (12.7%) |
|  |  | **Lack of remorse/guilt, lack of empathy, shallow/superficial emotions, uncaring attitudes about performance** | **92 (48.4%)** | **104 (67.1%)** | **42 (59.2%)** |
|  |  | Defiance towards authority figures, hostility, non-compliance, deceitfulness/lying, verbal and physical aggression | 13 (6.8%) | 5 (3.2%) | 1 (1.4%) |
| 34 | TRUE OR FALSE: Students with Conduct Problems and Limited Prosocial Emotions require tailored behavioural management strategies because they have unique deficits and needs. | **True** | **176 (96.7%)** | **142 (94.7%)** | **65 (94.2%)** |
|  |  | False | 6 (3.3%) | 8 (5.3%) | 4 (5.8%) |
| 35 | Which of the following options is NOT a relevant target behaviour for students with Limited Prosocial Emotions? | Caring behaviours (e.g., comforting others) | 54 (29.5%) | 20 (13.3%) | 17 (24.6%) |
|  |  | **Improved self-confidence** | **70 (38.3%)** | **91 (60.7%)** | **27 (39.1%)** |
|  |  | Recognising and responding to other’s distress | 23 (12.6%) | 12 (8.0%) | 11 (15.9%) |
|  |  | Reparative behaviours (e.g., apologising) | 36 (19.7%) | 27 (18.0%) | 14 (20.3%) |
| *Topic: Evidence-based principles of behavior management* | | |  | | |
| 4 | Select the correct word to fill in the blank. _______ means that a behaviour has been followed by a favourable consequence, and hence is more likely to happen again in the future. | Modelling | 16 (8.4%) | 8 (5.2%) | 8 (11.4%) |
|  |  | Shaping | 15 (7.9%) | 7 (4.6%) | 10 (14.3%) |
|  |  | **Reinforcement** | **152 (80.0%)** | **135 (88.2%)** | **52 (74.3%)** |
|  |  | Punishment | 7 (3.7%) | 3 (2.0%) | 0 (0.0%) |
| 5 | Select the correct word to fill in the blank. _______ means that a behaviour has been followed by an unfavourable consequence, and hence it is less likely to happen again in the future. | Modelling | 2 (1.1%) | 2 (1.3%) | 1 (1.4%) |
|  |  | Shaping | 14 (7.4%) | 3 (1.9%) | 6 (8.6%) |
|  |  | Reinforcement | 16 (8.4%) | 9 (5.8%) | 11 (15.7%) |
|  |  | **Punishment** | **158 (83.2%)** | **140 (90.9%)** | **52 (74.3%)** |
| 6 | Positive Reinforcement refers to: | **Introducing a pleasant consequence to increase a behaviour** | **177 (93.2%)** | **145 (94.2%)** | **59 (84.3%)** |
|  |  | Removing an unpleasant consequence to increase a behaviour | 8 (4.2%) | 2 (1.3%) | 4 (5.7%) |
|  |  | Introducing an unpleasant consequence to reduce a behaviour | 3 (1.6%) | 5 (3.2%) | 5 (7.1%) |
|  |  | Removing something pleasant to reduce a behaviour | 2 (1.1%) | 2 (1.3%) | 2 (2.9%) |
| 7 | Negative Reinforcement refers to: | Introducing a pleasant consequence to increase a behaviour | 2 (1.1%) | 3 (2.0%) | 3 (4.3%) |
|  |  | **Removing an unpleasant consequence to increase a behaviour** | **49 (26.1%)** | **79 (51.6%)** | **26 (37.1%)** |
|  |  | Introducing an unpleasant consequence to reduce a behaviour | 101 (53.7%) | 44 (28.8%) | 33 (47.1%) |
|  |  | Removing something pleasant to reduce a behaviour | 36 (19.1%) | 27 (17.6%) | 8 (11.4%) |
| 8 | Positive Punishment refers to: | Introducing a pleasant consequence to increase a behaviour | 36 (19.0%) | 23 (15.0%) | 11 (15.7%) |
|  |  | Removing an unpleasant consequence to increase a behaviour | 47 (24.9%) | 15 (9.8%) | 13 (18.6%) |
|  |  | **Introducing an unpleasant consequence to reduce a behaviour** | **55 (29.1%)** | **96 (62.7%)** | **31 (44.3%)** |
|  |  | Removing something pleasant to reduce a behaviour | 51 (27.0%) | 19 (12.4%) | 15 (21.4%) |
| 9 | Negative Punishment refers to: | Introducing a pleasant consequence to increase a behaviour | 2 (1.1%) | 4 (2.6%) | 2 (2.9%) |
|  |  | Removing an unpleasant consequence to increase a behaviour | 15 (8.0%) | 12 (7.8%) | 8 (11.4%) |
|  |  | Introducing an unpleasant consequence to reduce a behaviour | 98 (52.1%) | 39 (25.5%) | 21 (30.0%) |
|  |  | **Removing something pleasant to reduce a behaviour** | **73 (38.8%)** | **98 (64.1%)** | **39 (55.7%)** |
| 10 | Generally speaking, what are some of the functions of student misbehaviour? | A. To get attention, assistance, social interactions, control, or sensory stimulation | 11 (5.9%) | 7 (4.6%) | 4 (5.7%) |
|  |  | B. To avoid demands from others, social interactions, activities, or sensory stimulation | 1 (0.5%) | 5 (3.3%) | 2 (2.9%) |
|  |  | **Both A and B** | **173 (92.0%)** | **138 (90.2%)** | **62 (88.6%)** |
|  |  | None of the above | 3 (1.6%) | 3 (2.0%) | 2 (2.9%) |
| 11 | When trying to determine the function of a student’s problematic behaviour, what factors should you consider? | What happened before the problem behaviour (triggers) | 13 (6.9%) | 13 (8.5%) | 6 (8.6%) |
|  |  | What happened after the problem behaviour (reactions) | 0 (0.0%) | 1 (0.7%) | 1 (1.4%) |
|  |  | **What happened before (triggers) and after the problem behaviour (reactions)** | **173 (92.0%)** | **135 (88.2%)** | **60 (85.7%)** |
|  |  | None of the above | 2 (1.1%) | 4 (2.6%) | 3 (4.3%) |
| 12 | The type of attention students get when misbehaving is often: | A. Immediate | 29 (15.4%) | 16 (10.5%) | 8 (11.4%) |
|  |  | B. Exciting and highly emotional with lots of verbal and non-verbal signs | 3 (1.6%) | 5 (3.3%) | 1 (1.4%) |
|  |  | **Both A and B** | **152 (80.9%)** | **128 (83.7%)** | **59 (84.3%)** |
|  |  | None of the above | 4 (2.1%) | 4 (2.6%) | 2 (2.9%) |
| 13 | What are some of the ways adults accidentally reinforce or maintain problematic behaviour? | A. Providing inconsistent consequences for misbehaviour | 6 (3.2%) | 9 (5.9%) | 4 (5.7%) |
|  |  | B. Paying attention to attention-seeking misbehaviour | 2 (1.1%) | 6 (3.9%) | 4 (5.7%) |
|  |  | C. Not paying attention to instances of appropriate or prosocial behaviour | 1 (0.5%) | 0 (0.0%) | 0 (0.0%) |
|  |  | B and C | 14 (7.5%) | 20 (13.1%) | 11 (15.7%) |
|  |  | **All of the above** | **164 (87.7%)** | **118 (77.1%)** | **51 (72.9%)** |
| *Topic: Evidence-based strategies of behavior management* | | |  | |  |
| 14 | What is one of the most important things to consider when trying to establish a positive relationship with a student? | Increase the amount of positive attention you give to that student | 41 (21.9%) | 45 (29.4%) | 13 (18.6%) |
|  |  | Loosen the rules and expectations you have for that student | 0 (0.0%) | 2 (1.3%) | 1 (1.4%) |
|  |  | Reduce the amount of negative attention you give to that student | 5 (2.7%) | 2 (1.3%) | 2 (2.9%) |
|  |  | **Generally provide more positive attention than negative attention** | **63 (33.7%)** | **72 (47.1%)** | **29 (41.4%)** |
|  |  | Ensure there is a balance in the amount of positive to negative attention you give to that student | 78 (41.7%) | 32 (20.9%) | 25 (35.7%) |
| 15 | Which of the following statements is NOT true about child-led play? | Provides the child with a sense of autonomy and independence | 14 (7.5%) | 6 (3.9%) | 12 (17.1%) |
|  |  | Fosters a stronger adult-child relationship | 50 (26.7%) | 37 (24.2%) | 13 (18.6%) |
|  |  | **Teaches the child how to be a good leader** | **78 (41.7%)** | **86 (56.2%)** | **31 (44.3%)** |
|  |  | Provides the child with an opportunity to receive high-quality, positive attention | 45 (24.1%) | 24 (15.7%) | 14 (20.0%) |
| 16 | TRUE OR FALSE: Providing children with labelled and unlabelled praises are equally effective ways of increasing the likelihood of specific behaviours occurring again in the future. | True | 77 (41.4%) | 47 (30.9%) | 29 (41.4%) |
|  |  | **False** | **109 (58.6%)** | **105 (69.1%)** | **41 (58.6%)** |
| 17 | Which of the following factors are NOT characteristic of effective praise? | Delivered consistently and immediately after a desirable behaviour | 5 (2.7%) | 3 (2.0%) | 2 (2.9%) |
|  |  | Delivered enthusiastically | 8 (4.3%) | 4 (2.6%) | 6 (8.6%) |
|  |  | Specific in nature | 3 (1.6%) | 4 (2.6%) | 5 (7.1%) |
|  |  | **Used sparingly at first when teaching a new skill** | **170 (91.4%)** | **141 (92.8%)** | **57 (81.4%)** |
| 18 | What is the purpose of describing a child’s appropriate behaviour? | Shows that you are interested in their feelings and allows you to understand the child better | 30 (16.1%) | 14 (9.2%) | 6 (8.6%) |
|  |  | **Helps to keep the child’s attention on the task and shows that you approve of their behaviour** | **89 (47.8%)** | **86 (56.6%)** | **36 (51.4%)** |
|  |  | Models good language skills and provides an opportunity to place all the focus on the child and how they are feeling | 43 (23.1%) | 40 (26.3%) | 21 (30.0%) |
|  |  | None of the above | 24 (12.9%) | 12 (7.9%) | 7 (10.0%) |
| 19 | Which of the following is an effective way to describe an emotion (also known as emotion coaching)? | **You look sad from the way you are frowning and looking down** | **161 (87.5%)** | **137 (90.1%)** | **62 (88.6%)** |
|  |  | It is annoying you that the blocks keep falling down... | 20 (10.9%) | 9 (5.9%) | 5 (7.1%) |
|  |  | You’re jealous that your friend got to have a turn before you | 3 (1.6%) | 5 (3.3%) | 3 (4.3%) |
|  |  | You look like you are bored | 0 (0.0%) | 1 (0.7%) | 0 (0.0%) |
| 20 | Which of the following is the best example of a clear direction: | “Can you please pass me the crayons” | 6 (3.3%) | 3 (2.0%) | 2 (2.9%) |
|  |  | “Will you hand me the red block” | 26 (14.1%) | 18 (11.8%) | 9 (12.9%) |
|  |  | **“Please pass me the paintbrushes from the bucket”** | **122 (66.3%)** | **112 (73.7%)** | **56 (80.0%)** |
|  |  | “I’d like you to sit still please” | 30 (16.3%) | 19 (12.5%) | 3 (4.3%) |
| 21 | Which of the following is NOT a clear direction: | “Sit in your seat please” | 10 (5.4%) | 4 (2.6%) | 4 (5.7%) |
|  |  | **“Could you take out the art supplies please”** | **154 (83.7%)** | **132 (86.8%)** | **50 (71.4%)** |
|  |  | “Please pick up your rubbish” | 7 (3.8%) | 4 (2.6%) | 8 (11.4%) |
|  |  | “Put your books in a neat pile” | 13 (7.1%) | 12 (7.9%) | 8 (11.4%) |
| 22 | TRUE OR FALSE: Using questions during child-led play is a good way to show that you are interested. | TRUE | 173 (94.0%) | 117 (77.0%) | 57 (82.6%) |
|  |  | **FALSE** | **11 (6.0%)** | **35 (23.0%)** | **12 (17.4%)** |
| 23 | What is negative talk during child-led play? | A. Statements that express disapproval of the child or what s/he is doing | 3 (1.6%) | 3 (2.0%) | 3 (4.3%) |
|  |  | B. Correcting mistakes and providing constructive feedback | 0 (0.0%) | 0 (0.0%) | 1 (1.4%) |
|  |  | C. Sarcastic and/or rude statements towards the child | 11 (6.0%) | 5 (3.3%) | 3 (4.3%) |
|  |  | **All of the above** | **44 (23.9%)** | **60 (39.5%)** | **31 (44.9%)** |
|  |  | Options A and C | 126 (68.5%) | 84 (55.3%) | 31 (44.9%) |
| 24 | Why should negative talk be avoided? | A. Lowers child’s self-esteem | 8 (4.3%) | 4 (2.6%) | 3 (4.3%) |
|  |  | B. Increases the behaviour you want the child to stop | 3 (1.6%) | 2 (1.3%) | 3 (4.3%) |
|  |  | C. Models rude or inappropriate behaviour to the child | 0 (0.0%) | 2 (1.3%) | 0 (0.0%) |
|  |  | Both A and C | 69 (37.5%) | 46 (30.3%) | 22 (31.9%) |
|  |  | **All of the above** | **104 (56.5%)** | **98 (64.5%)** | **41 (59.4%)** |
| 25 | What types of rewards are most effective for motivating appropriate student behaviours? | **A. Social and tangible rewards depending on the child’s preferences** | **81 (44.3%)** | **74 (48.7%)** | **30 (43.5%)** |
|  |  | B. Social rewards (e.g., high-5, praise) | 7 (3.8%) | 2 (1.3%) | 2 (2.9%) |
|  |  | C. Tangible rewards (e.g., stickers, tokens) | 0 (0.0%) | 1 (0.7%) | 0 (0.0%) |
|  |  | Both B and C | 84 (45.9%) | 64 (42.1%) | 31 (44.9%) |
|  |  | None of the above, using rules and limits is the most effective way of motivating appropriate behaviours in students | 11 (6.0%) | 11 (7.2%) | 6 (8.7%) |
| 26 | Which of the following factors are NOT important when using rewards to motivate behaviour? | **Selection of rewards should be changed occasionally as a surprise to the child** | **140 (76.5%)** | **117 (77.0%)** | **55 (79.7%)** |
|  |  | Rewards should be provided immediately and consistently after appropriate behaviour occurs | 8 (4.4%) | 9 (5.9%) | 3 (4.3%) |
|  |  | Selection of rewards should be changed occasionally in consultation with the child | 25 (13.7%) | 16 (10.5%) | 5 (7.2%) |
|  |  | Having a clear idea of which behaviours warrant a reward versus those that do not | 10 (5.5%) | 10 (6.6%) | 6 (8.7%) |
| 28 | Which of the following options is NOT a characteristic of a clear direction? | Direct and specific | 0 (0.0%) | 2 (1.3%) | 3 (4.3%) |
|  |  | **Simultaneously explains why compliance is necessary** | **140 (76.5%)** | **126 (83.4%)** | **52 (75.4%)** |
|  |  | Developmentally appropriate | 12 (6.6%) | 9 (6.0%) | 6 (8.7%) |
|  |  | Polite and respectful | 31 (16.9%) | 14 (9.3%) | 8 (11.6%) |
| 29 | From the options below, select the direction that is BOTH positively stated AND direct: | “You need to stop being so silly” | 3 (1.6%) | 4 (2.6%) | 1 (1.4%) |
|  |  | “No yelling in the classroom” | 11 (6.0%) | 3 (2.0%) | 4 (5.8%) |
|  |  | “Can you use your inside voice” | 119 (65.0%) | 81 (53.6%) | 32 (46.4%) |
|  |  | **“Turn the computer off”** | **50 (27.3%)** | **63 (41.7%)** | **32 (46.4%)** |
| 30 | What is the most important thing to keep in mind BEFORE giving a clear direction? | **Consider if the child is actively listening and you have time to follow-through if the child does not comply** | **26 (14.2%)** | **32 (21.2%)** | **13 (18.8%)** |
|  |  | Consider if the direction has been followed in the past and if other students have struggled to comply too | 7 (3.8%) | 3 (2.0%) | 4 (5.8%) |
|  |  | Consider if the direction is necessary, if the child is actively listening, and is in a good emotional and physical state to be able to comply | 143 (78.1%) | 107 (70.9%) | 46 (66.7%) |
|  |  | None of the above | 7 (3.8%) | 9 (6.0%) | 6 (8.7%) |
| 31 | Which of the following is most effective when responding to student behaviour that functions to get the child out of following a direction: | **Validate the child’s feelings briefly, then re-direct to the original request. If needed, assist the child in following through with the request** | **165 (90.2%)** | **137 (91.3%)** | **59 (85.5%)** |
|  |  | Offer to compromise and make the direction a bit easier to follow | 10 (5.5%) | 7 (4.7%) | 2 (2.9%) |
|  |  | Send the child to time out for refusing to comply | 4 (2.2%) | 3 (2.0%) | 2 (2.9%) |
|  |  | Ignore the child’s non-compliance and wait for him/her to eventually follow the original direction | 4 (2.2%) | 3 (2.0%) | 6 (8.7%) |
| 32 | Bradley calls out the teacher’s name loudly in class while the teacher is assisting another student. It appears the function of Bradley’s behaviour is to get the teacher’s attention. What is the most effective way of responding to this type of attention-seeking behaviour? | A. Send Bradley to time out for shouting in class | 3 (1.6%) | 1 (0.7%) | 2 (2.9%) |
|  |  | B. Provide Bradley with multiple warnings and then begin placing an ‘X’ next to his name on the board. Each time he calls out, place an X next to his name and remind him that he will get detention if he does not stop shouting | 1 (0.5%) | 1 (0.7%) | 1 (1.4%) |
|  |  | C. Validate Bradley’s desire to get attention then remind him to raise his hand | 39 (21.3%) | 16 (10.7%) | 7 (10.1%) |
|  |  | D. Ignore Bradley’s behaviour and wait for him to stop shouting. Once quiet, praise him for engaging in a positive opposite behaviour (e.g., raising his hand, using quiet, calm voice, waiting patiently) | 7 (3.8%) | 11 (7.3%) | 4 (5.8%) |
|  |  | E. Ignore Bradley’s behaviour and praise positive behaviours in surrounding children (e.g., “I like how Lucy is raising her hand to speak and waiting patiently”) | 8 (4.4%) | 7 (4.7%) | 6 (8.7%) |
|  |  | Options B and D | 5 (2.7%) | 5 (3.3%) | 0 (0.0%) |
|  |  | **Options D and E** | **120 (65.6%)** | **109 (72.7%)** | **49 (71.0%)** |
| 33 | For a student that has just successfully  completed timeout and re-joined the class, why is it important to quickly find something positive in their behaviour to praise? | A. Helps to restore the student-teacher relationship | 9 (4.9%) | 1 (0.7%) | 4 (5.8%) |
|  |  | B. Highlights to the student that positive behaviour is rewarded with attention while misbehaviour results in removal of attention (e.g., timeout) | 13 (7.1%) | 21 (14.0%) | 6 (8.7%) |
|  |  | C. Builds the child’s self-esteem back up | 7 (3.8%) | 3 (2.0%) | 1 (1.4%) |
|  |  | **Options A and B** | **76 (41.5%)** | **58 (38.7%)** | **27 (39.1%)** |
|  |  | Option B and C | 78 (42.6%) | 67 (44.7%) | 31 (44.9%) |

*Note.* Correct answer is indicated in bold.

**Supplementary Table 2**

*Strategy Implementation Survey Items and Ratings of how Frequently each Strategy is Used by Overall Educator Sample*

| **Item** | **Time Point** | **Rating Frequency (%)** | | | | |
| --- | --- | --- | --- | --- | --- | --- |
| **Recommended classroom management strategy** |  | **Never** | **Rarely** | **Sometimes** | **Often** | **Very Often** |
| Praise | Pre | 1 (0.5) | 0 (0.0) | 15 (8.2) | 65 (35.7) | 101 (55.5) |
|  | Post | 1 (0.7) | 1 (0.7) | 9 (6.0) | 53 (35.3) | 86 (57.3) |
|  | 5WFU | 0 (0.0) | 0 (0.0) | 4 (5.9) | 28 (41.2) | 36 (52.9) |
| Tokens/Stickers for appropriate behaviour | Pre | 15 (8.2) | 7 (3.8) | 44 (24.2) | 61 (33.5) | 55 (30.2) |
|  | Post | 13 (8.7) | 10 (6.7) | 31 (20.7) | 52 (34.7) | 44 (29.3) |
|  | 5WFU | 3 (4.4) | 7 (10.3) | 16 (23.5) | 25 (36.8) | 17 (25.0) |
| Awards/Certificates | Pre | 21 (11.5) | 8 (4.4) | 60 (33.0) | 57 (31.3) | 36 (19.8) |
|  | Post | 14 (9.3) | 11 (7.3) | 45 (30.0) | 52 (34.7) | 28 (18.7) |
|  | 5WFU | 3 (4.4) | 8 (11.8) | 24 (35.3) | 22 (32.4) | 11 (16.2) |
| Prizes | Pre | 31 (17.0) | 19 (10.4) | 54 (29.7) | 49 (26.9) | 29 (15.9) |
|  | Post | 26 (17.3) | 17 (11.3) | 44 (29.3) | 37 (24.7) | 26 (17.3) |
|  | 5WFU | 9 (13.2) | 16 (23.5) | 20 (29.4) | 10 (14.7) | 13 (19.1) |
| Time Out (e.g. Sit & Watch) | Pre | 30 (16.5) | 52 (28.6) | 64 (35.2) | 24 (13.2) | 12 (6.6) |
|  | Post | 37 (24.7) | 47 (31.3) | 39 (26.0) | 23 (15.3) | 4 (2.7) |
|  | 5WFU | 17 (25.0) | 12 (17.6) | 24 (35.3) | 9 (13.2) | 6 (8.8) |
| Planned Ignoring | Pre | 44 (24.2) | 28 (15.4) | 57 (31.3) | 33 (18.1) | 20 (11.0) |
|  | Post | 12 (6.6) | 20 (13.3) | 53 (35.3) | 48 (32.0) | 17 (11.3) |
|  | 5WFU | 8 (11.8) | 6 (8.8) | 20 (29.4) | 24 (35.3) | 10 (14.7) |
| Validate & Redirect | Pre | 1 (0.5) | 4 (2.2) | 50 (27.5) | 84 (46.2) | 43 (23.6) |
|  | Post | 3 (2.0) | 7 (4.7) | 46 (30.7) | 62 (41.3) | 32 (21.3) |
|  | 5WFU | 3 (4.4) | 2 (2.9) | 15 (22.1) | 36 (52.9) | 12 (17.6) |
| **Non-Recommended Classroom management strategies** |  | **Never** | **Rarely** | **Sometimes** | **Often** | **Very Often** |
| Detentions | Pre | 46 (25.3) | 68 (37.4) | 51 (28.0) | 15 (8.2) | 2 (1.1) |
|  | Post | 50 (33.3) | 48 (32.0) | 38 (25.3) | 14 (9.3) | 0 (0.0) |
|  | 5WFU | 26 (38.2) | 21 (30.9) | 16 (23.5) | 4 (5.9) | 1 (1.5) |
| Sending student to principal’s office | Pre | 109 (59.9) | 43 (23.6) | 23 (12.6) | 6 (3.3) | 1 (0.5) |
|  | Post | 96 (64.0) | 35 (23.3) | 15 (10.0) | 4 (2.7) | 0 (0.0) |
|  | 5WFU | 41 (60.3) | 13 (19.1) | 9 (13.2) | 5 (7.4) | 0 (0.0) |
| Repeated verbal warnings | Pre | 4 (2.2) | 9 (4.9) | 54 (29.7) | 74 (40.7) | 41 (22.5) |
|  | Post | 5 (3.3) | 15 (10.0) | 38 (25.3) | 66 (44.0) | 26 (17.3) |
|  | 5WFU | 4 (5.9) | 8 (11.8) | 21 (30.9) | 20 (29.4) | 15 (22.1) |
| Removal of stockers/tokens for misbehaviour | Pre | 134 (73.6) | 27 (14.8) | 14 (7.7) | 6 (3.3) | 1 (0.5) |
|  | Post | 110 (73.3) | 27 (18.0) | 7 (4.7) | 5 (3.3) | 1 (0.7) |
|  | 5WFU | 50 (73.5) | 11 (16.2) | 3 (4.4) | 2 (2.9) | 2 (2.9) |
| School Suspensions | Pre | 120 (65.9) | 20 (11.0) | 34 (18.7) | 5 (2.7) | 3 (1.6) |
|  | Post | 94 (62.7) | 24 (16.0) | 21 (14.0) | 10 (6.7) | 1 (0.7) |
|  | 5WFU | 43 (63.2) | 10 (14.7) | 9 (13.2) | 5 (7.4) | 1 (1.5) |

*Note.* The Strategy Implementation Survey is made up of recommended evidence-based classroom management skills, along with common classroom management skills that are not typically recommended*.* Each item is rated on a scale of 0 to 4. A ranking of 0 is ‘Never, while a ranking of 4 is ‘Very often’. Higher scores indicate higher frequency of skill usage. M = Mean. SD = Standard Deviation. *N* (Pre) = 182, *N* (Post) = 150, and *N* (5WFU) = 68.

**Supplementary Table 3**

*Reliability of Outcome Measures at Each Time Point*

| **Outcome Measure** | **Time Point** | **Cronbach’s Alpha** | **McDonald’s Omega** |
| --- | --- | --- | --- |
| Knowledge | Pre | .71 | .71 |
|  | Post | .82 | .82 |
|  | 5WFU | .91 | .91 |
| Self-Efficacy | Pre | .86 | .86 |
|  | Post | .90 | .91 |
|  | 5WFU | .92 | .92 |
| Student-Teacher Closeness ^a^ | Pre | .84 | .85 |
|  | Post | .86 | .86 |
|  | 5WFU | .90 | .90 |
| Student-Teacher Conflict ^a^ | Pre | .82 | .82 |
|  | Post | .81 | .81 |
|  | 5WFU | .82 | .83 |
| Student Conduct Problems ^a^ | Pre | .69 | .70 |
|  | Post | .68 | .70 |
|  | 5WFU | .78 | .79 |
| Student Callous-Unemotional Traits ^a^ | Pre | .87 | .87 |
|  | Post | .90 | .90 |
|  | 5WFU | .93 | .93 |
| Frequency of Use of Recommended Classroom Management Strategies | Pre | .69 | .71 |
|  | Post | .73 | .73 |
|  | 5WFU | .71 | .72 |
| Utility of Recommended Classroom Management Strategies | Pre | .86 | .87 |
|  | Post | .85 | .85 |
|  | 5WFU | .93 | .93 |
| Frequency of Use of Non-Recommended Classroom Management Strategies | Pre | .65 | .65 |
|  | Post | .69 | .69 |
|  | 5WFU | .74 | .75 |
| Utility of Non-Recommended Classroom Management Strategies | Pre | .69 | .71 |
|  | Post | .73 | .74 |
|  | 5WFU | .83 | .87 |
| Acceptability | Pre |  |  |
|  | Post | .95 | .95 |
|  | 5WFU |  |  |
| Feasibility | Pre |  |  |
|  | Post | .94 | .94 |
|  | 5WFU |  |  |

*Note*. Pre = Pre-intervention (baseline questionnaire that participants complete before being randomly assigned to a condition); Post = Post-intervention (the questionnaire that participants complete immediately after the iPCMT modules); 5WFU = 5-week follow-up (the final questionnaire that is administered to participants 5 weeks after the completing iPCMT). Knowledge = 35-item knowledge test; Self-Efficacy = 4-item Classroom Management subscale from the Teacher’s Sense of Self-Efficacy Scale-Short Form (TSSE-SF); Student Conduct Problems = 5-item conduct problems subscale within the Strengths and Difficulties Questionnaire (SDQ); Student Callous-Unemotional Traits = 8-item shortened version of the Inventory of Callous-Unemotional Traits. Frequency of Recommended Classroom Management Strategies = 7-item subscale within frequency portion of the Strategy Implementation Survey. Utility of Recommended Classroom Management Strategies = 7-item subscale within the usefulness portion of the Strategy Implementation Survey. Frequency of Non-Recommended Classroom Management Strategies = 5-item subscale within frequency portion of the Strategy Implementation Survey. Utility of Non-Recommended Classroom Management Strategies = 5-item subscale within the usefulness portion of the Strategy Implementation Survey. Acceptability = 5-item Acceptability of Intervention Measure. Feasibility = 4-item Feasibility of Intervention Measure.

^a^ Only inclusive of participants who nominated a disruptive student by providing their initials at the beginning of the study. 161 participants within the intent-to-treat sample provided student initials.

**Supplementary Table 4**

*Demographic Characteristics of Educators for Total Sample and Completer Status*

| Variable | Total Sample | Completers | Drop-Outs | Test Statistics |
| --- | --- | --- | --- | --- |
|  | *M (SD)* | *M (SD)* | *M (SD)* | *t or* χ2 |
| Educator Age (years) | *N* = 228  40.22 (12.51) | *n* = 64  43.45 (13.13) | *n* = 164  38.96 (12.06) | *t*(226*)* = 2.46, *p* = .02* |
|  | *N (%)* | *N (%)* | *N (%)* |  |
| Condition | *N* = 228 | *N* = 64 | *N* = 164 | χ2(1) = 6.61, *p* = .01*^a^ |
| Immediate-iPCMT | 115 (50.4) | 41 (64.1) | 74 (45.1) |  |
| Waitlist Control | 113 (49.6) | 23 (35.9) | 90 (54.9) |  |
| Gender | *N* = 204 | *N* = 54 | *N* = 150 | χ2(1) = 3.77, *p* = .052^b^ |
| Woman | 177 (86.8) | 51 (94.4) | 126 (84.0) |  |
| Man | 26 (12.7) | 3 (5.6) | 23 (15.3) |  |
| Prefer not to answer | 1 (0.5) | 0 (0.0) | 1 (0.7) |  |
| Race / Ethnicity | *N* = 228 | *N* = 64 | *N* = 164 | χ2(1) = .19, *p* = .67^c^ |
| White | 165 (72.4) | 45 (70.3) | 120 (73.2) |  |
| Asian | 21 (9.2) | 8 (12.5) | 13 (7.9) |  |
| Pacific Islander | 2 (0.9) | 0 (0.0) | 2 (1.2) |  |
| Middle Eastern | 14 (6.1) | 2 (3.1) | 12 (7.3) |  |
| Aboriginal or Torres Strait Islander | 6 (2.6) | 2 (3.1) | 4 (2.4) |  |
| African | 2 (0.9) | 1 (1.6) | 1 (0.6) |  |
| Other | 18 (7.9) | 6 (9.4) | 12 (7.3) |  |
| Current Role | *N* = 104 | *N* = 33 | *N* = 71 | χ2(1) = .71, *p* = .40^d^ |
| Teacher | 72 (69.2) | 21 (63.6) | 51 (71.8) |  |
| Learning Support Staff or Paraprofessional | 20 (19.2) | 8 (24.2) | 12 (16.9) |  |
| Other | 12 (11.5) | 4 (12.1) | 8 (11.3) |  |
| Years Experience | *N* = 227 | *N* = 64 | *N* = 163 | χ2(1) = 6.16, *p* = .01*^e^ |
| 0-5 years (early) | 78 (34.4) | 14 (21.9) | 64 (39.3) |  |
| 6-15 years (mid) | 76 (33.5) | 28 (43.8) | 48 (29.4) |  |
| 16-30+ years (established) | 73 (32.2) | 22 (34.4) | 51 (31.3) |  |
| Accreditation Status | *N* = 227 | *N* = 64 | *N* = 163 | χ2(1) = 6.12, *p* = .01*^f^ |
| Unaccredited | 14 (6.2) | 5 (7.8) | 9 (5.5) |  |
| Conditionally accredited | 15 (6.6) | 1 (1.6) | 14 (8.6) |  |
| Provisionally accredited | 45 (19.8) | 7 (10.9) | 38 (23.3) |  |
| Proficient | 150 (66.1) | 51 (79.7) | 99 (60.7) |  |
| Highly accomplished | 2 (0.9) | 0 (0.0) | 2 (1.2) |  |
| Lead | 1 (0.4) | 0 (0.0) | 1 (0.6) |  |
| Employment Status | *N* = 227 | *N* = 64 | *N* = 163 | χ2(1) = 1.68, *p* = .20^g^ |
| Permanent full-time | 112 (49.3) | 33 (51.6) | 79 (48.5) |  |
| Permanent part-time | 18 (7.9) | 8 (12.5) | 10 (6.1) |  |
| Temporary full-time | 67 (29.5) | 15 (23.4) | 52 (31.9) |  |
| Temporary part-time/Casual | 28 (12.3) | 8 (12.5) | 20 (12.3) |  |
| Other | 2 (0.9) | 0 (0.0) | 2 (1.2) |  |
| Geographic Location | *N* = 228 | *N* = 64 | *N* = 164 | χ2(1) = 0.13, *p* = .72^h^ |
| Metropolitan | 204 (89.5) | 58 (90.6) | 146 (89.0) |  |
| Regional | 21 (9.2) | 6 (9.4) | 15 (9.1) |  |
| Rural | 3 (1.3) | 0 (0.0) | 3 (1.8) |  |
| Type of School | *N* = 227 | *N* = 64 | *N* = 163 | *p* = .69^i^ |
| Government/public | 219 (96.5) | 61 (95.3) | 158 (96.9) |  |
| Systemic (e.g., Catholic) | 2 (0.9) | 1 (1.6) | 1 (0.6) |  |
| Independent/Non-government | 5 (2.2) | 2 (3.1) | 3 (1.8) |  |
| Other | 1 (0.4) | 0 (0.0) | 1 (0.6) |  |
| Institution Setting | *N* = 226 | *N* = 64 | *N* = 162 | *p* = 1.00^j^ |
| Early childhood | 1 (0.4) | 0 (0.0) | 1 (0.6) |  |
| Primary/Elementary | 225 (99.6) | 64 (100.0) | 161 (99.4) |  |

*Note.* **p* < .05. M = Mean. SD = Standard Deviation.

^a^Condition binarized (1 = Immediate-iPCMT; 2 = Waitlist Control) for chi-squared test.

^b^Gender binarized (1 = Woman; 2 = Man, Non-Binary) for chi-squared test.

^c^Race binarized (1 = White, 2 = Asian, Middle Eastern, Aboriginal or Torres Strait Islander, African, Pacific Islander, Other) for chi-squared test.

^d^Current Role binarized (1 = Teacher, 2 = Learning Support, Paraprofessional) for chi-squared test.

^e^Years experience binarized (1 = Early, 2 = Mid, Established) for chi-squared test.

^f^Accreditation Status binarized (1 = Unaccredited, Conditionally accredited, Provisionally accredited, 2 = Proficient, Highly accomplished, Lead) for chi-squared test.

^g^Employment Status binarized (1 = Permanent full-time, Temporary full-time, 2 = Permanent part-time, Temporary part-time/Casual) for chi-squared test.

^h^Geographic location binarized (1 = Metropolitan, 2 = Rural, Regional) for chi-squared test.

^i^Type of School (1 = Government/Public, 2 = Systemic, Independent/Non-government, Other) for Fisher’s Exact test.

^j^Institution Setting (1 = Primary/Elementary, 2 = Early childhood) for Fisher’s Exact test.

**Supplementary Table 5**

*Descriptive Statistics and Comparisons Between Time Points for Overall Educator Sample*

| **Outcome Measure** | **Time Point** | ***n*** | **Mean (SD)** |
| --- | --- | --- | --- |
| Knowledge | Pre | 189 | 20.68 (4.45) |
|  | Post | 154 | 23.60 (5.50) |
|  | 5WFU | 77 | 19.66 (8.03) |
| Self-Efficacy | Pre | 183 | 29.51 (4.20) |
|  | Post | 150 | 30.22 (4.12) |
|  | 5WFU | 68 | 29.43 (4.54) |
| Student-Teacher Closeness ^a^ | Pre | 129 | 31.33 (6.19) |
|  | Post | 101 | 31.75 (6.13) |
|  | 5WFU | 47 | 31.22 (6.67) |
| Student-Teacher Conflict ^a^ | Pre | 129 | 23.68 (5.81) |
|  | Post | 101 | 22.47 (6.08) |
|  | 5WFU | 47 | 23.23 (5.70) |
| Frequency of Use of Recommended Classroom Management Strategies | Pre | 182 | 17.08 (4.59) |
|  | Post | 150 | 17.17 (4.67) |
|  | 5WFU | 68 | 17.34 (4.58) |
| Utility of Recommended Classroom Management Strategies | Pre | 175 | 17.98 (5.19) |
|  | Post | 145 | 19.70 (4.95) |
|  | 5WFU | 63 | 20.17 (5.67) |
| Frequency of Use of Non-Recommended Classroom Management Strategies | Pre | 182 | 5.65 (2.95) |
|  | Post | 150 | 5.31 (3.05) |
|  | 5WFU | 68 | 5.34 (3.58) |
| Utility of Non-Recommended Classroom Management Strategies | Pre | 145 | 8.05 (4.20) |
|  | Post | 113 | 8.75 (4.10) |
|  | 5WFU | 49 | 10.51 (4.75) |

*Note*. Pre = Pre-intervention (baseline questionnaire that participants complete before being randomly assigned to a condition); Post = Post-intervention (the questionnaire that participants complete immediately after the iPCMT modules); 5WFU = 5-week follow-up (the final questionnaire that is administered to participants 5 weeks after the completing iPCMT). Knowledge = 35-item knowledge test on conduct problems; Self-Efficacy = 4-item Classroom Management subscale from the Teacher’s Sense of Self-Efficacy Scale-Short Form; Student Conduct Problems = 5-item conduct problems subscale within the Strengths and Difficulties Questionnaire; Student Callous-Unemotional Traits = 8-item shortened version of the Inventory of Callous-Unemotional Traits. *n* = Number of educators who completed each outcome measure at a given time point.

^a^ Only inclusive of participants who nominated a disruptive student by providing their initials at the beginning of the study. 161 participants within the intent-to-treat sample provided student initials.

**Supplementary Table 6**

*Descriptive Statistics and Comparisons Between the Immediate-iPCMT and Wait-list Control Conditions for Educator Outcome Measures*

| **Outcome Measure** | **Time Point** | **Immediate-iPCMT condition** | | **Wait-list Control condition** | |
| --- | --- | --- | --- | --- | --- |
|  |  | ***n*** | **Mean (SD)** | ***n*** | **Mean (SD)** |
| Knowledge | Pre | 109 | 20.83 (4.10) | 112 | 19.97 (4.61) |
|  | Post | 91 | 24.16 (4.82) | 80 | 20.48 (4.91) |
| Self-Efficacy | Pre | 106 | 29.67 (4.17) | 106 | 29.69 (3.72) |
|  | Post | 90 | 30.61 (4.01) | 77 | 29.29 (4.26) |
| Student-Teacher Closeness ^a^ | Pre | 82 | 32.11 (5.89) | 71 | 30.44 (5.94) |
|  | Post | 65 | 32.09 (6.27) | 47 | 29.98 (6.53) |
| Student-Teacher Conflict ^a^ | Pre | 82 | 23.96 (5.14) | 71 | 24.44 (5.47) |
|  | Post | 65 | 22.58 (5.77) | 47 | 23.18 (6.86) |
| Student Conduct Problems ^a^ | Pre | 82 | 6.66 (2.22) | 70 | 6.46 (2.32) |
|  | Post | 65 | 6.56 (2.17) | 46 | 6.38 (2.51) |
| Student Callous-Unemotional Traits ^a^ | Pre | 81 | 26.14 (3.89) | 70 | 25.78 (4.51) |
|  | Post | 64 | 25.26 (4.35) | 46 | 26.05 (4.04) |

*Note*. Pre = Pre-intervention (where all participants, irrespective of condition, complete the baseline questionnaire before random assignment to a condition). Post = Post-intervention (for the Immediate-iPCMT condition, the questionnaire that was administered after finishing the iPCMT modules, whereas for the Waitlist Control condition, the repeat baseline questionnaire that was administered after the 11-week waiting period); Knowledge = 35-item knowledge test on conduct problems; Self-Efficacy = 4-item Classroom Management subscale from the Teacher’s Sense of Self-Efficacy Scale-Short Form; Student Conduct Problems = 5-item conduct problems subscale within the Strengths and Difficulties Questionnaire; Student Callous-Unemotional Traits = 8-item shortened version of the Inventory of Callous-Unemotional Traits. *n* = Number of educators who completed each outcome measure at a given time point.

^a^ Only inclusive of participants who nominated a disruptive student by providing their initials at the beginning of the study. 161 participants within the intent-to-treat sample provided student initials.
